# Elucidating the role of S100A10 in CD8^+^ T cell exhaustion and HCC immune escape via the cPLA2 and 5-LOX axis

**DOI:** 10.1038/s41419-024-06895-0

**Published:** 2024-08-08

**Authors:** Ganggang Wang, Xiaowei Shen, Wenzhi Jin, Chao Song, Meiyuan Dong, Zhijie Zhou, Xiaoliang Wang

**Affiliations:** 1 Department of Hepatobiliary Surgery, Shanghai Pudong Hospital, Fudan University Pudong Medical Center, 2800 Gongwei Road, Pudong, Shanghai, 201399 China; 2 Department of General Surgery, QingPu Branch of Zhongshan Hospital Affiliated to Fudan University, QingPu District Central Hospital Shanghai, No. 1158, Gong Yuan Dong Road, Shanghai, 201700 China; 3 Department of Pancreatic Surgery, Zhongshan Hospital, Fudan University, Shanghai, P. R. China; 4 Department of Endocrinology, Shanghai Pudong Hospital, Fudan University, Shanghai, People’s Republic of China

**Keywords:** Cancer, Diseases

## Abstract

Hepatocellular carcinoma (HCC) is a common malignant tumor with a complex immune evasion mechanism posing a challenge to treatment. The role of the S100A10 gene in various cancers has garnered significant attention. This study aims to elucidate the impact of S100A10 on CD8^+^ T cell exhaustion via the cPLA2 and 5-LOX axis, thereby elucidating its role in immune evasion in HCC. By analyzing the HCC-related data from the GEO and TCGA databases, we identified differentially expressed genes associated with lipid metabolism and developed a prognostic risk model. Subsequently, through RNA-seq and PPI analyses, we determined vital lipid metabolism genes and downstream factors S100A10, ACOT7, and SMS, which were significantly correlated with CD8^+^ T cell infiltration. Given the most significant expression differences, we selected S100A10 for further investigation. Both in vitro and in vivo experiments were conducted, including co-culture experiments of CD8^+^ T cells with MHCC97-L cells, Co-IP experiments, and validation in an HCC mouse model. S100A10 was significantly overexpressed in HCC tissues and potentially regulates CD8^+^ T cell exhaustion and lipid metabolism reprogramming through the cPLA2 and 5-LOX axis. Silencing S100A10 could inhibit CD8^+^ T cell exhaustion, further suppressing immune evasion in HCC. S100A10 may activate the cPLA2 and 5-LOX axis, initiating lipid metabolism reprogramming and upregulating LTB4 levels, thus promoting CD8^+^ T cell exhaustion in HCC tissues, facilitating immune evasion by HCC cells, ultimately impacting the growth and migration of HCC cells. This research highlights the critical role of S100A10 via the cPLA2 and 5-LOX axis in immune evasion in HCC, providing new theoretical foundations and potential targets for diagnosing and treating HCC.

## Introduction

Hepatocellular carcinoma (HCC) is one of the most prevalent malignant tumors worldwide and represents the predominant form of liver cancer [1, 2]. This type of cancer is primarily linked to chronic infections of hepatitis B and C viruses, alcoholic liver disease, and non-alcoholic fatty liver disease [3]. Diagnosing and treating HCC have always posed challenges in the medical field [4]. Owing to the absence of clear symptoms during the initial stages, a considerable number of patients have already advanced to later stages when diagnosed, thereby restricting treatment alternatives and prognosis [5]. Furthermore, the variability of HCC, the complex nature of liver anatomy, and the invasive characteristics of tumors further exacerbate the challenges in treatment [6]. Immune evasion plays a pivotal role in the advancement of liver cancer [7]. Tumors hamper the immune system’s response by employing different mechanisms, for example, by expressing immune suppressive factors that dampen the activity of T cells [8]. However, this limitation has not only hindered the effectiveness of traditional immunotherapy but has also opened doors for the development of novel treatment approaches, such as inhibitors that specifically target the PD-1/PD-L1 pathway [9, 10]. Although there are challenges in treating and diagnosing the condition, a comprehensive understanding of the underlying mechanisms, particularly immune escape, has yielded novel therapeutic approaches and instilled optimism [11].

The integration of bioinformatics with RNA transcriptome sequencing (RNA-seq) is a powerful tool in biomedical research, enabling comprehensive identification of gene expression patterns to investigate disease mechanisms and therapeutic targets. In hepatocellular carcinoma (HCC), RNA-seq effectively identifies genetic abnormalities and novel transcriptional alterations [12–14]. It provides crucial clues for an in-depth understanding of the occurrence, development, and metastasis of HCC [15]. By implementing bioinformatics methods, the present study predicted a putative involvement of S100A10 in HCC. Moreover, this gene could potentially be linked to immune escape in liver cancer, suggesting its involvement in suppressing the immune response and facilitating tumor growth.

Nevertheless, the role of S100A10 in the immune evasion of HCC remains unclear. According to prior literature, an interaction has been observed between S100A10 and cPLA2 proteins [16, 17]. cPLA2 plays a vital role in the transmission of cellular signals. When activated improperly, it can contribute to the development of numerous cancers [18]. Therefore, integrating bioinformatics and RNA-seq technology has elucidated the fundamental molecular mechanisms underlying HCC and pinpointed S100A10 as a promising therapeutic target. Future investigations into the interaction between S100A10 and cPLA2 could yield novel avenues and approaches for treating HCC.

The research aims to investigate the role of S100A10 in HCC, specifically its impact on immune evasion mechanisms. Building on the aforementioned conclusions, the objective of this study is to elucidate the relationship between S100A10 and the cPLA2 and 5-LOX axis and its role in inducing exhaustion of CD8^+^ T cells. This research is significant because it provides a thorough understanding of how S100A10 promotes immune evasion in HCC. Consequently, it contributes to the identification of novel therapeutic approaches, especially in immune-based treatments for HCC.

## Materials and Methods

### Human liver cell lines and in vitro culture

The human normal liver cell line MIHA (catalog: bio-132746), human hepatic stellate cell line LX-2 (catalog: bio-77172), highly metastatic human HCC cell line HCCLM3 (catalog: bio-73219), human HCC cell line Huh-7 (catalog: bio-73061), human HCC cell line MHCC97-L (catalog: Bio-73438), and the mouse HCC cell line Hepa 1-6 (Catalog: bio-53546) were procured from Biobw (China Microbial Culture Collection Center, Beijing, China). MIHA and MHCC97-L represent normal human liver cells, whereas HCCLM3, Huh-7, and MHCC97-L are human HCC cells. These distinct cell lines were employed to assess the expression of vital genes associated with lipid metabolism, among other factors. Cultivation of the cells was carried out in DMEM (catalog number: 11965092, Gibco), supplemented with 20% fetal bovine serum (FBS, catalog number: 12483020, Gibco), 2 mM L-glutamine (catalog number: A2916801, Gibco), 100 units/mL penicillin, and 100 µg/mL streptomycin (catalog number: 15140148, Gibco). The cells were maintained at 37 °C within a 5% carbon dioxide incubator. These reagents were procured from Thermo Fisher (USA) [19].

### Retrieval of TCGA and GEO data

The TCGA-HCC transcriptome data and clinical data, along with the GSE76427 chip transcriptome data and clinical data, were obtained from the TCGA database (https://portal.gdc.cancer.gov/) and the GEO database (https://www.ncbi.nlm.nih.gov/), respectively. Perl scripts were applied, and organizational procedures were employed to acquire gene matrix and clinical data files. Within the TCGA-HCC dataset, there were a total of 424 human liver samples, comprising 50 normal and 374 HCC samples. Similarly, the GSE76427 dataset encompassed 167 human liver samples, consisting of 52 normal and 115 HCC samples. A collection of genes associated with lipid metabolism was retrieved from the KEGG PATHWAY Database website (https://www.kegg.jp/kegg/pathway.html) and subjected to analysis using the R software packages “limma” and “sva” to identify differentially expressed genes related to lipid metabolism that were common to both the TCGA and GSE76427 datasets. Subsequently, the expression profiles of genes associated with lipid metabolism were merged with clinical data from the intersection of the TCGA and GSE76427 datasets [20].

Subsequently, a Lasso regression model for prognosis analysis was constructed using TCGA data as the training set and the GSE76427 chip data as the validation set. By utilizing the “survival” and “glmnet” packages in R language, a risk-scoring model was initially established based on the expression levels of lipid metabolism-related genes. This model calculates the risk score for each patient by summing the weighted values of each gene’s expression with its Lasso regression coefficient. Patients were then categorized into high- and low-risk groups by comparing their risk scores to the median: those with risk scores above the median were placed in the high-risk group, while those equal to or below the median were assigned to the low-risk group. The performance of the model was evaluated using R software packages including, “limma,” “ggplot2,” “survminer,” “pheatmap,” “timeROC,” “ggpubr,” “ComplexHeatmap,” “reshape2,” “GSVA,” and “GSEABase.” This evaluation involved Principal Component Analysis (PCA), overall survival analysis, Cox regression analysis, progression-free survival (PFS) analysis, risk factor analysis, receiver operating characteristic (ROC) analysis, and clinical trait correlation analysis. The differences in immune cells, immune functions, immune checkpoint genes, and immunotherapy sensitivity between high-risk and low-risk groups were analyzed. The set of immune checkpoint genes was obtained by literature review [21], and the immunotherapy scoring file was obtained from the TIDE website (http://tide.dfci.harvard.edu/).

### RNA transcriptome sequencing (RNA-seq)

Three normal tissue samples were procured via in vitro cultivation of the human liver cell line MIHA, while three tumor tissue samples were similarly acquired through in vitro cultivation of the human HCC cell line MHCC97-L. Subsequently, the total RNA isolation from all six samples was carried out employing the Total RNA Isolation Reagent Kit (catalog number: 12183555, Invitrogen, USA). The quantification of total RNA was achieved by measuring the OD value, and the integrity of these total RNAs was assessed using the agarose gel electrophoresis method. High-quality total RNA was then subjected to reverse transcription to generate cDNA, followed by the construction of RNA libraries and sequencing on the Illumina NextSeq 500 platform. The initial sequencing data were obtained in the form of raw reads, following the conversion of raw image data through base calling. Subsequently, Cutadapt was utilized to eliminate sequencing adapter sequences and filter out low-quality sequences, thereby ensuring the quality of the raw reads. The remaining reads were denoted as “clean reads.” These clean reads were aligned to the human reference genome using the Hisat2 software, and gene expression quantification was performed utilizing the R software package to generate the gene expression matrix. Differential expression analysis was carried out using the “edgeR” R package, employing a filtering threshold of |log2FC | > 1 and *p* < 0.05. To further refine the results, they were intersected with a gene set pertaining to lipid metabolism [20], to identify differentially expressed genes specifically associated with lipid metabolism.

### Further screening of genes related to key lipid metabolism

The next step involved the intersection of the differentially expressed genes associated with lipid metabolism, as identified through RNA-seq analysis, with the set of lipid metabolism-related genes employed in the construction of a prognostic risk model. Subsequently, survival analysis was conducted on the genes resulting from this intersection using the R package “survival.” To gain further insights into the significance of these genes, they were input into the TIMER2.0 website (http://timer.comp-genomics.org/) to assess their correlation with CD8^+^ T cells in HCC, aiming to pinpoint essential lipid metabolism-related genes. Furthermore, the proteins encoded by the differentially expressed genes related to lipid metabolism, as obtained from the sequencing data, were submitted to the String website (https://cn.string-db.org/). A confidence level of 0.75 was set, and proteins with no binding partners were excluded to construct the protein-protein interaction (PPI) network [20].

### Acquisition of CD8^+^ T cells in humans

Peripheral blood samples were procured from both healthy individuals and patients diagnosed with HCC during routine health assessments. The isolation of peripheral blood mononuclear cells (PBMCs) from these samples was carried out using Ficoll-Hypaque (Cat No: 10771, Sigma, USA). Additionally, HCC tissue specimens were collected from HCC patients. These blood and tissue samples were subsequently employed for the isolation and research of CD8^+^ T cells. The extraction and purification of CD8^+^ T cells were achieved utilizing the CD8^+^ T Cell Isolation Kit (Catalog number: 11348D, Invitrogen) [22]. Following isolation, the CD8^+^ T cells were preserved in RPMI 1640 medium (Catalog No. 11875101, Gibco, USA), supplemented with 30 U/ml of IL-2 (Catalog No. PHC0021, Gibco). Flow cytometry analysis was conducted on the separated cells to assess their purity. A purity level exceeding 95% was considered necessary to meet the experimental criteria [23]. All chemicals and reagents used in this study were purchased from Sigma (Germany) and Thermo Fisher (USA) [24]. Furthermore, the experimental program was granted approval by our hospital’s ethics committee, and informed consent was obtained from both the healthy participants and HCC patients involved in the study.

### Interaction between Human CD8^+^ T Cells and LTB4 in vitro

CD8^+^ T cells isolated from HCC patient HCC tissues were cultured under 37 °C, 5% CO_2_ conditions for 48 h. Dynabeads™ CD3/CD28 (Catalog number: 11161D, ThermoFisher, USA) were added to the culture medium to expand and activate T cells. Additionally, 10 μM and 20 μM of LTB4 (Catalog number: L0517, Sigma, Germany) were separately added to the culture medium. After 48 h, flow cytometry was used to assess the status of CD8^+^ T cells and validate the effect of CD8^+^ T cell depletion molecules [25].

### Construction of orthotopic xenograft models

A total of thirty BALB/C nude mice, aged between 4 and 6 weeks and weighing 15-25 grams, were procured from Shanghai SLAC Laboratory Animal Co., Ltd. (Shanghai, China). These nude mice were accommodated in a sterile SPF-grade animal facility and underwent a one-week acclimatization period during which their health and condition were monitored [26]. The experimental protocol and procedures involving the use of animals were duly reviewed and approved by our hospital’s Institutional Animal Care and Use Committee. To conduct the study, the thirty nude mice were randomly divided into two groups: the normal group and the tumor group, each consisting of 15 mice. The procedure commenced with the anesthetization of the nude mice through inhalation of isoflurane, followed by their secure positioning in a supine posture. Subsequently, after iodine disinfection, the abdominal cavity was carefully opened to expose the liver, and 1 × 10^6^ MHCC97-L cells were gradually injected into the liver. Following the injection, the abdominal cavity was sealed, and 2 × 10^6^ human CD8^+^ T cells were introduced via the tail vein. The progression of in situ HCC tumors was monitored using live animal imaging, and the nude mice were humanely euthanized when the tumor attained a maximum diameter of 3–5 mm. Following euthanasia, the liver was collected and utilized for further experimental investigations, including the isolation of CD8^+^ T cells [22].

### Isolation and detection of CD8^+^ T cells from nude mice

Following the establishment of the xenograft tumor model in the aforementioned nude mice, we acquired 10 samples of normal liver tissue and 10 samples of tumor liver tissue for further analysis. These tissue specimens were meticulously sectioned into pieces measuring 1-2 mm in size using scissors. Subsequently, they underwent treatment with a mixture containing 30 μg/ml DNase I (catalog number: D7076, Beyotime, China), 0.1 mg/ml hyaluronidase (catalog number: ST1384, Beyotime, China), and 1 mg/ml collagenase (catalog number: ST2303, Beyotime, China) at room temperature, while gently agitating for a duration of 2 h. Following this treatment, the resulting cell suspension was filtered through a 70 μm mesh to obtain a single-cell suspension [22]. This prepared single-cell suspension was carefully layered onto 10 ml of Ficoll-Paque (product number: 10771, Sigma) and centrifuged at 400 g, 20 °C for 30 min. After centrifugation, the intermediate cloudy layer, consisting of lymphocytes, was further separated using a CD8^+^ T cell isolation kit (product number: 11348D, Invitrogen). This process allowed us to isolate CD8^+^ T cells from the nude mice for subsequent experimental investigations. The cells obtained from the separation process were subjected to treatment with anti-CD8^+^ T cell antibodies (catalog numbers: 344722, 344706, Biolegend, USA), and their purity was evaluated using flow cytometry. A purity level exceeding 95% was deemed necessary to meet the experimental criteria [23]. The above materials were purchased from Beyotime (China), Sigma (Germany), and Thermofisher (USA).

### Cell transfection

The silencing plasmid targeting S100A10, along with its corresponding lentiviral vectors, were generously provided by Hanheng Biotechnology Co., Ltd. (Shanghai, China). Each lentiviral vector was introduced into the cells at a multiplicity of infection (MOI) of 10, with the concurrent presence of 5 µg/mL puromycin (Catalogue number: A1113803, Gibco, USA) for selection purposes [19]. The cell transfection experiments encompassed four distinct groups: the Control group, the S100A10-sh-NC group, the S100A10-sh-1 group, and the S100A10-sh-2 group (Table S1-2).

### Co-culture of HCC cells and CD8^+^ T cells in vitro

The isolated CD8^+^ T cells were subjected to co-culture with either transfected or non-transfected MHCC97-L cells at a ratio of 1:5, under controlled conditions of 37°C and 5% CO_2_, for a duration of 48 h. To facilitate T cell expansion and activation, Dynabeads™ CD3/CD28 (part number: 11161D, Thermofisher, USA) were introduced into the culture medium. In the co-culture system, CD8^+^ T cells were placed in the upper chamber, while MHCC97-L cells were seeded in the lower chamber of a Transwell plate (catalog number: CLS3412, Sigma-Aldrich, Germany). A 0.4 µm membrane separated the CD8^+^ T cells and MHCC97-L cells, allowing only soluble factors to pass through. Following the 48-hour co-culture period, both the supernatant and the cultured cells were collected for subsequent experiments [23].

The in vitro co-culture experiments involved the following groups: Con group, S100A10-sh-NC group, S100A10-sh group, S100A10-sh+MT group, and S100A10-sh+MT + ZT group. The cPLA2 selective agonist Melittin (MT, Cat. No: S9794) and 5-LOX selective inhibitor Zileuton (ZT, Cat. No: S1443) were procured from Selleck (China). Melittin was administered at a concentration of 1 μM, while Zileuton was applied at 5 μM. Both compounds were introduced into the culture medium of MHCC97-L cells at the initiation of co-culture, with an incubation period of 48 h [27].

### MTT assay

Following the manufacturer’s guidelines, the co-cultured MHCC97-L cells were harvested, resuspended, and enumerated. Subsequently, these cells were seeded at a density of 2 × 10^3^ cells per well in a 96-well plate. To assess the proliferation of MHCC97-L cells, the Cell Proliferation Assay Kit (Catalog: C0009S, Beyotime, China) was employed [19].

### Co-IP assay

Cells were subjected to lysis using lysis buffer (catalog: P0013F, Beyotime, China), and subsequent removal of cellular debris was achieved through centrifugation. The resulting sample was collected, and the concentration of each sample was adjusted to an equivalent level. A portion of the sample was reserved as the Input, while the remaining supernatant, matched in terms of concentration and volume, was subjected to incubation with either an anti-S100A10 antibody (#SAB1409624, Sigma, 1:30) or an anti-cPLA2 antibody (#SAB4200210, Sigma, 1:30), along with protein A/G beads (#88302, Thermofisher, USA), for a duration of 2 h. Following incubation, the sample was subjected to three washes with a lysis buffer, followed by boiling the beads at 100 °C for 5 min. Subsequently, proteins were separated using sodium dodecyl sulfate-polyacrylamide gel electrophoresis, transferred onto nitrocellulose membranes (catalog number: HATF00010, Millipore), and subsequently subjected to immunoblotting [28]. The above materials were purchased from Beyotime (China), Sigma (Germany), and Thermofisher (USA).

### HPLC assay

The quantification of leukotriene B4 (LTB4) in the extracellular environment of MHCC97-L cells was carried out utilizing the reverse-phase high-performance liquid chromatography (HPLC) method. To summarize, the lower layer of the co-cultured clear liquid, obtained following pre-treatment, was employed for the determination of extracellular LTB4 content in the MHCC97-L cells by means of sample injection. The mobile phase used in the analysis consisted of a mixture of acetonitrile, water, and formic acid, with proportions ranging from 20:80:0.01 to 60:40:0.01. It’s important to note that the mobile phase gradient did not exceed 20 ml per gradient [29]. The LTB4 standard product (item number: L0517) used in this analysis was procured from Sigma (Germany).

### Validation of the association between LTB4 and CD8^+^ T cells in vitro

The isolated CD8^+^ T cells underwent a culture period at 37 °C with a 5% CO_2_ atmosphere, lasting for 48 h. During this incubation, Dynabeads™ CD3/CD28 (catalog number: 11161D, Thermofisher, USA) were introduced into the culture medium to facilitate the amplification and activation of T cells. Additionally, two concentrations of LTB4, namely 10 μM and 20 μM (Catalog number: L0517, Sigma, Germany), were supplemented into the culture medium. After the 48-hour incubation period, flow cytometry was employed to assess the condition of CD8^+^ T cells, thereby validating the involvement of effector molecules associated with CD8^+^ T cell exhaustion [25]. MHCC97-L cells transfected with S100A10 NC and S100A10-sh plasmids were cultured with isolated CD8^+^ T cells at 37 °C and 5% CO_2_ for 48 h. Dynabeads™ CD3/CD28 (product number: 11161D, Thermofisher, USA) were added to the culture medium to expand and activate the T cells. Flow cytometry was employed after 48 h to assess the status of CD8^+^ T cells and validate the molecular effects of CD8^+^ T cell depletion [25].

### In vivo experiments on tumor and CD8^+^ T cells in NOD-SCID mice with silencing S100A10

A total of 36 male NODs.CB17-Prkdcscid/NcrCrl mice, aged between 4-6 weeks and weighing 15–25 grams, were procured from Beijing Vital River Laboratory Animal Technology Co., Ltd. (Beijing, China). These mice, known as NOD-SCID mice, were accommodated in a specific pathogen-free (SPF) animal laboratory and underwent a 1-week acclimatization period during which their health and condition were monitored [19, 30, 31]. Approval for the experimental protocol and animal use procedures was granted by the Institutional Animal Care and Use Committee of Pudong Hospital, Fudan University.

The NOD-SCID mice were employed for the modeling process, which involved the injection of either MHCC97-L cells or transfected MHCC97-L cells into their livers. Concurrently, isolated human CD8^+^ T cells were intravenously administered via the tail vein. Depending on the type of MHCC97-L cells used, the NOD-SCID mice were categorized into the following groups, each containing 12 mice: control (con) group, S100A10-sh-NC group, and S100A10-sh group. To monitor the metastasis of cancer cells, fluorescence enzyme activity bioluminescence imaging was conducted utilizing the in vivo imaging system (Visque® InVivo Elite, Vieworks). Following anesthesia of the NOD-SCID mice with 2% isoflurane, two photographs were taken with an exposure time of 10 min, and observations were made at five-day intervals. Thirty days later, the NOD-SCID mice were euthanized, and their xenografts were photographed and measured [32].

### Effects of Silencing S100A10 on tumor and CD8^+^ T cell in BALB/c mice

Thirty-six male BALB/c mice aged 4–6 weeks (15–25 g) were obtained from Beijing Vital River Laboratory Animal Technology Co., Ltd. (Beijing, China) and were housed in SPF level animal facilities. The mice were acclimatized for one week, and their condition was observed. This experimental protocol and animal usage were approved by our hospital’s Animal Ethics Committee [33].

The mice were intravenously injected with Hepa 1-6 or transfected Hepa 1-6 cells to induce tumor growth in the liver. Based on the different types of Hepa 1-6 cells, the mice were divided into the following groups, each consisting of 12 mice: control (con) group, S100A10-sh-NC group, and S100A10-sh group. After 30 days, the mice were euthanized, and CD8^+^ T cells were isolated from the tumor tissues [33].

### Flow cytometry

To determine the total cell count and concentration of isolated T cells, a cell counter was employed. For cell surface staining, the cells were suspended in a 2% PBS solution and subsequently exposed to the following antibodies for flow cytometry analysis: anti-CD279 (PD-1, catalog number: 557946, BD), anti-TIM3 (catalog number: 565558, BD), anti-CD223 (LAG3, catalog number: 369307, BioLegend), anti-CTLA4 (catalog number: 369606, BioLegend), anti-Ki67 (catalog number: 558616, BD), and anti-CD69 antibody (catalog number: 310904, BioLegend). This incubation process occurred at 0 °C for a duration of 30 min, followed by two washes with 2% PBS and subsequent flow cytometry analysis. For intracellular staining, the cells underwent fixation and permeabilization before incubation with directly conjugated anti-IL-2 antibody (catalog number: 500310, BioLegend), anti-IFN-γ antibody (catalog number: 502515, BioLegend), and anti-TNF-α antibody (catalog number: 559321, BD) at 4 °C for 30 min. Flow cytometry was then performed to detect the stained cells. T cells requiring cytokine detection were initially stimulated with a cell stimulation mixture (catalog number: 00-4975-93, Thermofisher, USA) at 37 °C for 6 h before undergoing the appropriate staining procedure. The flow cytometry analysis of cells was executed utilizing the FACS Aria II Cell Sorter (BD Biosciences), and subsequent data analysis was carried out using FlowJo software (TreeStar) [22]. The above materials were purchased from BD (USA), BioLegend (USA), and Thermofisher (USA).

In the flow cytometry analysis of BALB/c mice, the following antibodies were added for flow cytometry detection: anti-Ki67 (catalog number: 558616, BD) and anti-CD69 antibody (catalog number: 104505, BioLegend). The cells were incubated on ice for 30 min, then washed twice with 2% PBS before flow cytometric analysis. For intracellular staining, cells were first fixed and permeabilized, then incubated at 4 °C with directly labeled fluorescent antibodies against IL-2 (catalog number: 503809, BioLegend), IFN-γ (catalog number: 505815, BioLegend), and TNF-α (catalog number: 561063, BD) for 30 min, followed by flow cytometric analysis.

### CD8^+^ T cell absolute count detection

The quantification of CD8^+^ T cells was achieved through flow cytometry. Initially, a gating analysis was conducted to isolate CD8^+^ T cells. Blood or cell samples were incubated with an anti-CD8 antibody (catalog number: ab256296, Abcam, UK) at 4 °C for 30 min. Subsequently, red blood cells were lysed, and white blood cells were obtained through PBS washing. The flow cytometry analysis of cells was performed utilizing the FACS Aria II Cell Sorter (BD Biosciences), and subsequent data analysis was conducted using FlowJo software (TreeStar) [34].

### RT-qPCR

The extraction of total RNA from both cells and liver tissue was initiated by homogenizing 100 mg of tissue in 1 mL of TRIzol reagent (catalog number: 10296010, Thermofisher, USA). Following this, 200 µL of chloroform was added to the mixture, ensuring thorough mixing. The resultant mixture was then subjected to centrifugation at 4 °C and 12,000 g for a duration of 10 min. The upper aqueous phase was collected and combined with isopropanol (500 µL) to induce RNA precipitation. The isolated RNA was subsequently dissolved in RNase-free water (10-30 µL) and quantified using a Nanodrop instrument (Nanodrop 3300, Thermofisher, USA) [35]. The reverse transcription of RNA into cDNA was carried out in accordance with the guidelines provided by TaqMan Reverse Transcription Reagents (part number: N8080234, Thermofisher, USA). PCR analysis was subsequently performed using the PowerUp SYBR Green Master Mix (catalog number: A25741, Thermofisher, USA). GAPDH was employed as an internal control to assess the relative expression levels of various genes, employing the 2^-△△CT^ method [36]. The primer sequences are shown in Table S3-4.

### Western blot

Total protein extraction from both cells and liver tissue was carried out using RIPA lysis buffer supplemented with 4% protease inhibitor (Catalog number: P0013B, BiyunTian, China) in accordance with the provided instructions, with a ratio of 1 mL per 100 mg of tissue. Protein concentration was determined using the BCA Protein Assay Kit (Cat. No. P0010S, Beyotime, China). Subsequently, equal quantities of protein samples were loaded onto an SDS-PAGE gel containing sodium dodecyl sulfate (SDS) and acrylamide for separation. The separated proteins were then transferred onto a PVDF membrane (catalog number: FFP24, Beyotime, China) using a wet transfer method. The PVDF membrane was subjected to incubation with TBST containing 5% skim milk (P0216-300 g, Beyotime, China) at room temperature for 1 h, followed by overnight incubation with the respective primary antibodies at 4 °C. The antibodies used in this study included anti-S100A10 (#SAB1409624, 1:500, Sigma), anti-cPLA2 (#5249, 1:1000, CST), anti-p-cPLA2 (#SAB420021, 1:1000, Sigma), anti-5-LOX (#SAB1410449, 1:1000, Sigma), anti-p-5-LOX (#3748, 1: 1000, CST), anti-ACOT7 (ab156576, 1:1000, Abcam, UK), anti-Spermine synthase (anti-SMS, ab156879, 1:1000, Abcam, UK), and anti-GAPDH antibody (#PA1-987, 1:1000, Thermofisher, USA). The anti-GAPDH antibody was procured from Thermofisher (USA), while the other primary antibodies mentioned above were obtained from Sigma (Germany). Following a TBST wash, the membrane underwent incubation with horseradish peroxidase-conjugated goat anti-rabbit secondary antibody (Catalog No: A0208, Beyotime, China) at room temperature for 1 h. Subsequently, the membrane was treated with the ECL chemiluminescent reagent kit (Catalog No: P0018FS, Beyotime, China) and visualized using the ChemiDoc XRS+ chemiluminescence detection system (Bio-Rad). The quantification of proteins was performed using ImageJ software, with comparisons made between the grayscale values of different proteins and the grayscale ratio of the internal reference protein, GAPDH [37]. Each experiment was repeated three times.

### Transmission electron microscopy

CD8^+^ T cells were initially fixed in a 2.5% paraformaldehyde solution (catalog number: 8.20603, Sigma) at a temperature of 25 °C for a duration of 1 h. Following this, they were further fixed with 1% osmium tetroxide/1.5% potassium ferricyanide (catalog number: P3289, Sigma) for an additional hour. Subsequent steps involved multiple washes and dehydration of the cells using acetone (catalog number: 48358, Sigma), followed by embedding in epoxy resin (catalog number: 45345, Sigma). Ultra-thin sections measuring 50 nm were prepared using the Leica Ultra (Leica Microsystems), and these sections were subsequently restained with vanadium chloride hematoxylin negative stain (catalog number: A51037, ThermoFisher). Microscopic images were captured using the Philips CM100 (ThermoFisher) transmission electron microscope, with an acceleration voltage set to 80 kV and the utilization of the TemCam-F416 digital camera (TVIPS). Image analysis and quantification were carried out using ImageJ software. This analysis was used to quantify the number of mitochondria within each cell, enabling the detection of mitochondrial damage and facilitating other related experiments [38]. The above materials were purchased from Sigma (Germany) and Thermofisher (USA).

### Mitochondrial membrane potential and quality assessment

CD8^+^ T cells underwent separate staining procedures with 10 nM MitoTracker Deep Red (MDR, Cat# M22426, ThermoFisher) and 100 nM MitoTracker Green (MG, Cat# M46750, ThermoFisher) at a temperature of 37 °C for a duration of 15 min. This staining protocol enabled the measurement of both mitochondrial membrane potential and total mass. The mitochondrial membrane potential was expressed in millivolts per unit mass (MDR/MG). Flow cytometry analysis was conducted to detect mitochondrial damage and perform other relevant experiments using the LSRII flow cytometer (BD Biosciences). Subsequently, data analysis was carried out using FlowJo software [38]. The materials above were purchased from Thermofisher (USA).

### Determination of mitochondrial DNA (mtDNA) copy number

Total DNA extraction was performed utilizing the Genome-tip 20/G kit (Catalog number: 10223, QIAGEN, USA). Subsequently, the copy number of mtRNA was quantified for experiments pertaining to the detection of mitochondrial damage and related studies, employing the mtDNA detection primer kit (Catalog number: 7246, Takara, Japan) [39].

### Statistical analysis

The analysis of all data was carried out utilizing SPSS 21.0 statistical software (IBM, USA). Continuous data were expressed as mean ± standard deviation. A t-test was employed to compare two groups, while a one-way analysis of variance (ANOVA) was utilized to compare multiple groups. Significance levels were denoted as follows: *p* < 0.05, *p* < 0.01, and *p* < 0.001, indicating statistically significant differences.

## Results

### S100A10 is potentially associated with the exhaustion of CD8^+^ T cells induced by HCC

Recent studies have shown that cancer cells could reprogram their lipid metabolism to create favorable conditions for their proliferation, ultimately leading to the exhaustion of CD8^+^ T cell function within the tumor microenvironment [40]. Based on the relationship between lipid metabolism reprogramming and CD8^+^ T cells, to investigate the genes associated with CD8^+^ T cell exhaustion induced by HCC, we downloaded transcriptomic and clinical data of HCC from TCGA and analyzed to obtain 219 differentially expressed genes related to lipid metabolism (Figure S1A).

**Fig. 1:**
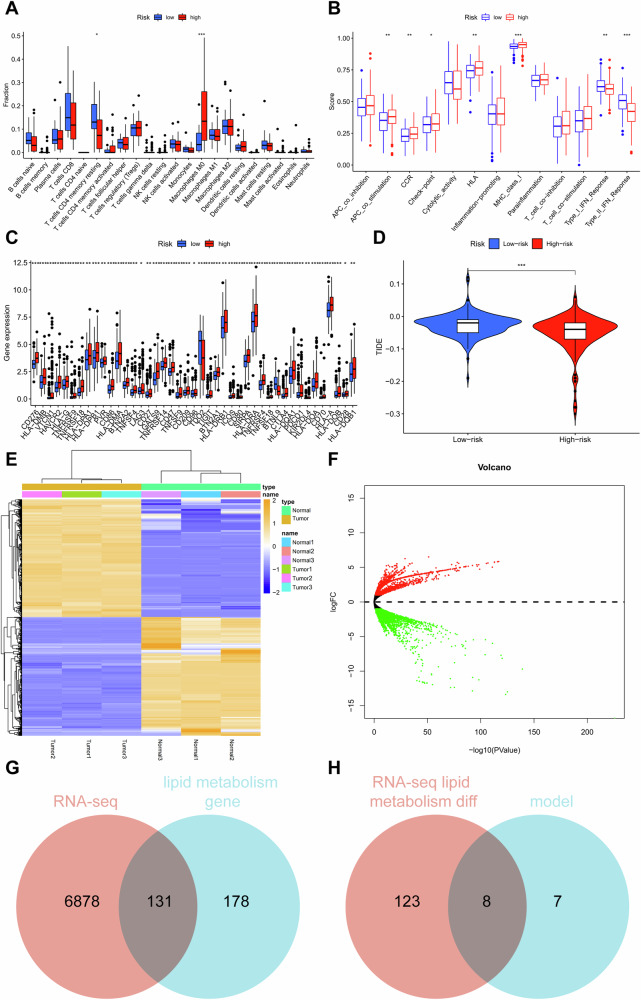
Analysis of immune differences and RNA high-throughput sequencing between high-risk and low-risk groups of HCC patients. **A** Analysis of immune cell infiltration differences between high-risk and low-risk groups of HCC patients in the TCGA database; **B** Analysis of immune function differences between high-risk and low-risk groups of HCC patients in the TCGA database; **C** Analysis of differential expression of immune checkpoint genes between high-risk and low-risk groups of HCC patients in the TCGA database; **D** Analysis of differential sensitivity to immune therapy between high-risk and low-risk groups of HCC patients in the TCGA database; **E** Heatmap of differentially expressed genes between normal liver cells and HCC cells detected by RNA-seq; (**F**) Volcano plot of differentially expressed genes between normal liver cells and HCC cells detected by RNA-seq, with red representing upregulation and green representing downregulation; **G** Venn diagram depicting the intersection between differentially expressed genes identified by RNA-seq and genes related to lipid metabolism; **H** Venn diagram depicting the intersection between differentially expressed genes related to lipid metabolism identified by RNA-seq and model genes. Each group in RNA-seq consists of 3 samples, * indicates comparison between two groups, *p* < 0.05, *p* < 0.01, **p < 0.001.

By extracting the common parts of differentially expressed genes related to lipid metabolism from the GSE76427 dataset and TCGA, we merged the obtained results with their clinical data and constructed a prognostic model using Lasso regression. We identified 15 key lipid metabolism genes, including ACOT7, S100A10, and APEX1 (Figure S1B-C, Table S5). We calculated the risk value by measuring the expression levels of 15 key genes involved in fatty acid metabolism in different samples of HCC patients. We divided all samples into high and low-risk groups based on the risk value and evaluated the model’s performance using methods such as Principal Component Analysis (PCA), survival analysis, Progression-Free Survival (PFS), and risk curve. We also validated the model using the GSE76427 dataset, and the results consistently showed that the model had a good prognostic effect on HCC patients (Figure S1D-L). Subsequently, by analyzing the correlation between the high-risk and low-risk groups with immune cells, we found significant differences between the high-risk and low-risk groups regarding immune cell infiltration, immune function, and expression levels of immune checkpoint genes (Fig. 1A-C). In the context of immunotherapy, it was noted that the TIDE score was higher in the low-risk group compared to the high-risk group. This suggests that patients in the low-risk group are more prone to immunologic escape, potentially leading to reduced efficacy of immunotherapy. It is speculated that individuals in the high-risk group may be better candidates for immunotherapy (Fig. 1D).

To further identify critical genes involved in lipid metabolism reprogramming in HCC, we obtained three normal samples from in vitro cultured MIHA liver cells and three HCC samples from MHCC97-L cells and performed RNA-seq high-throughput sequencing and differential analysis. We obtained 131 differentially expressed genes related to lipid metabolism (Fig. 1E-G). Therefore, we intersected the 131 differentially expressed genes in lipid metabolism obtained from RNA-seq with the 15 differentially expressed genes in lipid metabolism used to construct the Lasso model and identified 8 key genes: S100A10, PRDX6, APEX1, SMS, ACSL3, ACOT7, PRKAA2, and ME1 (Fig. 1H).

**Fig. 2:**
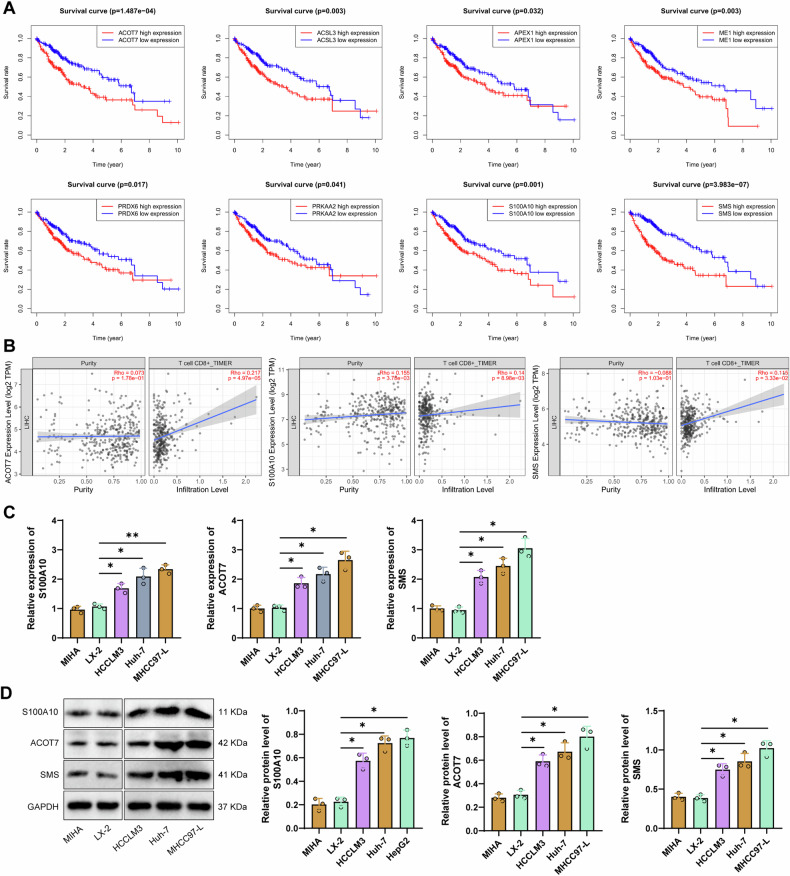
Correlation of S100A10 with survival time and CD8^+^ T cells in HCC patients. **A** Correlation between the expression levels of 8 lipid metabolism-related genes in tumor tissues of HCC patients and their survival time (*p* < 0.01); **B** Prediction of the correlation between 6 lipid metabolism-related genes and CD8^+^ T cells in HCC using TIMER2.0; **C, D** Detection of the mRNA and protein expression levels of S100A10, ACOT7, and SMS in 2 normal liver cells (MIHA, LX-2) and 3 HCC cells (HCCLM3, Huh-7, MHCC97-L) by RT-qPCR and Western blot, respectively. Cell experiments were repeated 3 times, * indicates comparison between two groups, *p* < 0.01, *p < 0.001.

Subsequently, we further analyzed the correlation between those above 8 key lipid metabolism genes and patient survival time and CD8^+^ T cell infiltration. Results showed that these 8 genes were significantly correlated with patient survival time (all *p* < 0.01), while only S100A10, ACOT7, and SMS were significantly associated with CD8^+^ T cell infiltration (Fig. 2A-B). Furthermore, we further examined the mRNA levels and protein expression of S100A10, ACOT7, and SMS in two types of normal liver cells (MIHA, LX-2) and three types of HCC cells (HCCLM3, Huh-7, MHCC97-L). The results showed that compared to normal liver cells, the mRNA and protein expression of S100A10, ACOT7, and SMS were significantly upregulated in HCC cells, with the most significant difference observed in S100A10 (Fig. 2C-D). S100A10 has been reported to be highly expressed in various cancer tissues, including HCC, and promotes the proliferation and migration of cancer cells [41–43].

Therefore, we believe that S100A10 may be a key factor in HCC-induced CD8^+^ T cell exhaustion.

### CD8^+^ T cells are depleted in HCC tissues

CD8^+^ T cells are the key performers of adaptive anti-tumor immunity, and their function relies on the metabolism of intracellular fatty acid (FA) molecules [44, 45]. In tumor tissues, the FAs in CD8^+^ T cells are extensively consumed, resulting in functional exhaustion characterized by reduced IFN-γ production and high expression of programmed cell death protein-1 (PD-1) in infiltrating CD8^+^ T cells [46, 47].

**Fig. 3:**
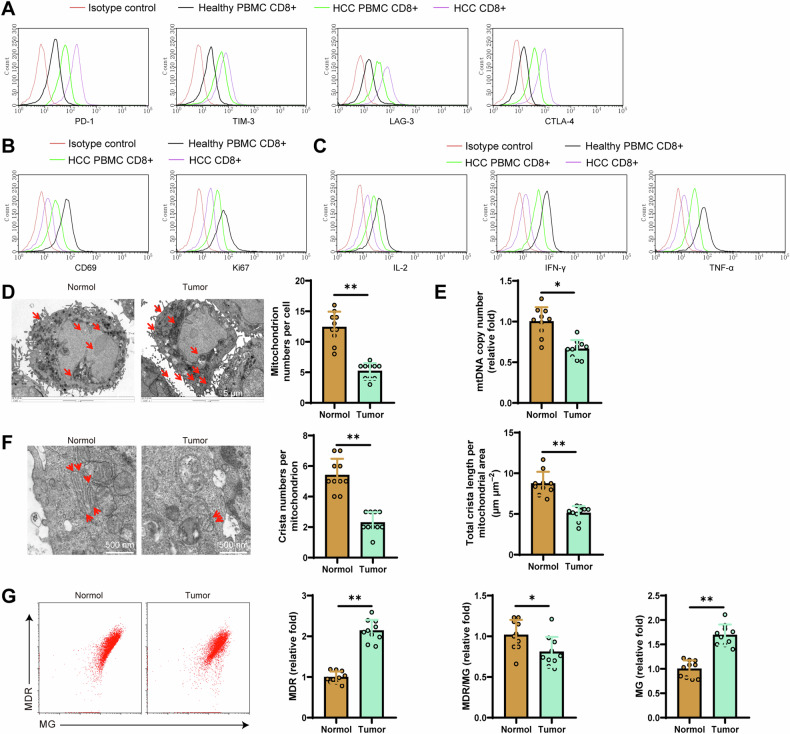
Determination of CD8^+^ T cell cellular status and mitochondrial damage. **A** Flow cytometry detection of the expression levels of depletion markers PD1, TIM3, LAG3, CTLA4, etc., in CD8^+^ T cells from each group; **B** Flow cytometry detection of the expression levels of Ki67 and CD69 in CD8^+^ T cells from each group; **C** Flow cytometry detection of the expression levels of IFN-γ, IL-2, and TNF-α in CD8^+^ T cells from each group; **D** Transmission electron microscopy observation of the number of mitochondria in CD8^+^ T cells, with red arrows indicating mitochondria, scale bar = 5 μm; **E** Detection of mtRNA copy numbers by RT-qPCR; **F** Transmission electron microscopy observation of the number and total length of mitochondrial cristae in CD8^+^ T cells, with red arrows indicating internal cristae, scale bar=500 nm; **G** Flow cytometry detection of the mitochondrial membrane potential in CD8^+^ T cells. Each group consists of 10 nude mice, * indicates comparison between two groups, *p* < 0.05, **p < 0.001.

We investigated whether CD8^+^ T cells in HCC tissues exhibit exhaustion using flow cytometry. The results showed that compared to healthy individuals’ PBMC CD8^+^ group, the expression levels of exhaustion markers such as PD1, TIM3, LAG3, and CTLA4 were significantly increased in HCC patients’ PBMC CD8^+^ group and HCC tissues’ CD8^+^ group. Additionally, their proliferative ability (Ki67) and cell vitality (CD69) were weakened, and the levels of IFN-γ, IL-2, and TNF-α production were decreased (Fig. 3A-C). Therefore, we speculate that CD8^+^ T cells are depleted in HCC tissues.

Subsequently, by constructing the xenograft tumor model in situ, we extracted CD8^+^ T cells from the liver tissue and peripheral blood of both the Normal group and the Tumor group nude mice for purity and absolute quantification analysis (purity >96%, Figure S2). Furthermore, it has also been reported that CD8^+^ T cells in a state of functional exhaustion exhibit evident mitochondrial damage [48–50]. Therefore, we assessed the extent of CD8^+^ T cell mitochondrial damage in the liver tissues of nude mice’s Normal and Tumor groups. Mitochondria play crucial and diverse roles in different stages of T cell adaptive responses, and a decrease in mitochondrial quantity signifies T cell functional suppression and exhaustion [51]. The results revealed a significant reduction in the number of mitochondria in CD8^+^ T cells from the Tumor group compared to the Normal group, accompanied by a notable decrease in mtRNA copy numbers, internal cristae quantity and length, as well as membrane potential (Fig. 3D-G).

### Silencing of S100A10 inhibits the exhaustion of CD8^+^ T cells induced by MHCC97-L cells

**Fig. 4:**
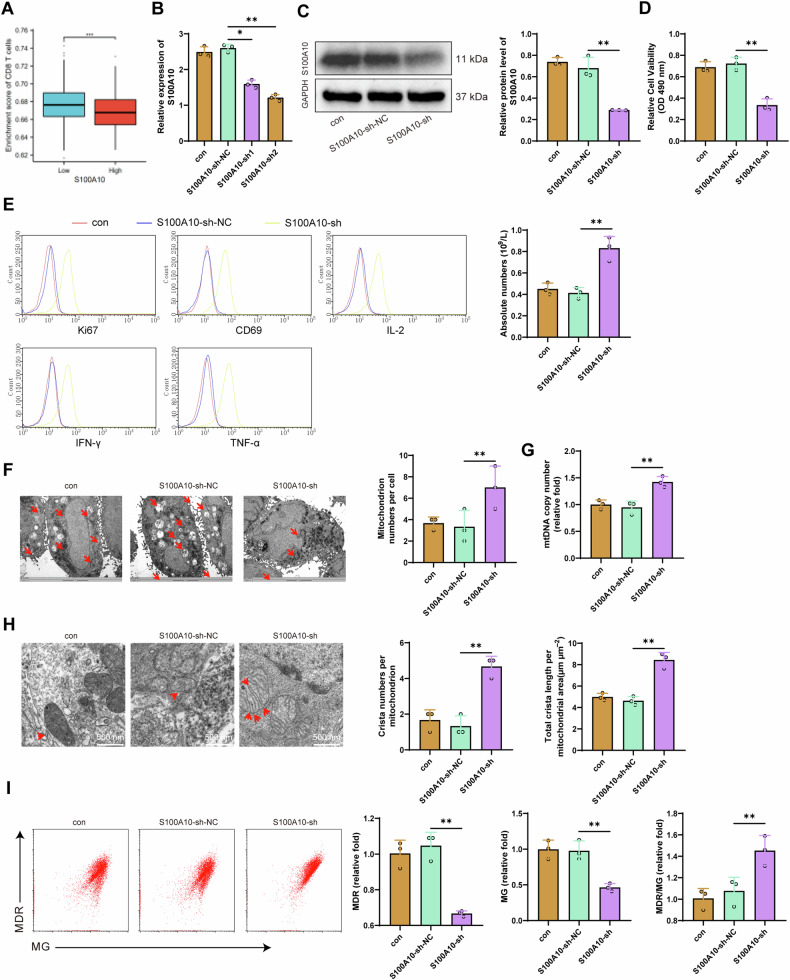
Effects of silencing S100A10 on HCC-induced CD8^+^ T cell exhaustion. **A** CD8^+^ T cell infiltration in S100A10; (**B**) Detection of S100A10 mRNA expression levels in each group of cells by RT-qPCR; **C** Detection of S100A10 protein expression levels in MHCC97-L cells by Western blot in each group; **D** MTT assay to detect cell proliferation in each group of MHCC97-L cells; **E** Flow cytometry detection of the expression levels of Ki67, CD69, IL-2, IFN-γ, and TNF-α, and the absolute number of CD8^+^ T cells in each group; **F** Transmission electron microscopy observation of the number of mitochondria in CD8^+^ T cells, with red arrows indicating mitochondria, scale bar=5 μm; **G** Detection of mtRNA copy numbers in CD8^+^ T cells by RT-qPCR; **H** Transmission electron microscopy observation of the number and total length of mitochondrial cristae in CD8^+^ T cells, with red arrows indicating internal cristae, scale bar=500 nm; **I** Flow cytometry detection of mitochondrial quality and membrane potential in CD8^+^ T cells. Cell experiments were repeated 3 times, * indicates comparison between two groups, *p* < 0.05, *p* < 0.01, **p < 0.001.

To further investigate the correlation between S100A10 and CD8^+^ T cell exhaustion induced by HCC, we observed a significant decrease in CD8^+^ T cell infiltration in the group with high S100A10 expression compared to the group with low S100A10 expression (Fig. 4A). This finding suggests that S100A10 may facilitate the exhaustion of CD8^+^ T cells in the HCC microenvironment. In subsequent experiments, we designed a specific silencing sequence for S100A10 and transfected it into MHCC97-L cells. We selected the sequence with the most effective silencing for further experiments (Fig. 4B).

By co-culturing transfected MHCC97-L cells with CD8^+^ T cells and using Transwell as a medium, we promote a cell culture environment that closely mimics natural conditions [52]. We investigated the impact of silencing S100A10 on CD8^+^ T cell exhaustion. Western blot and MTT results showed that compared with the S100A10-sh-NC group, the viability of MHCC97-L cells in the S100A10-sh group decreased, and the expression of S100A10 protein in the cells was significantly reduced (Fig. 4C-D). Flow cytometry analysis revealed that compared to the S100A10-sh-NC group, the S100A10-sh group exhibited enhanced proliferation capacity (Ki67) and cell vitality (CD69) of CD8^+^ T cells. Additionally, the IFN-γ, IL-2, and TNF-α production levels increased. Furthermore, there was an increase in the absolute number of CD8^+^ T cells (Fig. 4E).

Furthermore, transmission electron microscopy results revealed a significant increase in the number of mitochondria in CD8^+^ T cells of the S100A10-sh group compared to the S100A10-sh-NC group, along with a marked increase in both the quantity and length of internal cristae (Fig. 4F, Fig. 4H). RT-qPCR results demonstrated that compared to the S100A10-sh-NC group, the CD8^+^ T cell mtRNA copy number significantly increased in the S100A10-sh group (Fig. 4G). Flow cytometry analysis demonstrated that compared with the S100A10-sh-NC group, silencing S100A10 inhibited the decrease of mitochondrial membrane potential in CD8^+^ T cells (Fig. 4I).

### S100A10 induces CD8^+^ T cell exhaustion through the cPLA2 and 5-LOX

**Fig. 5:**
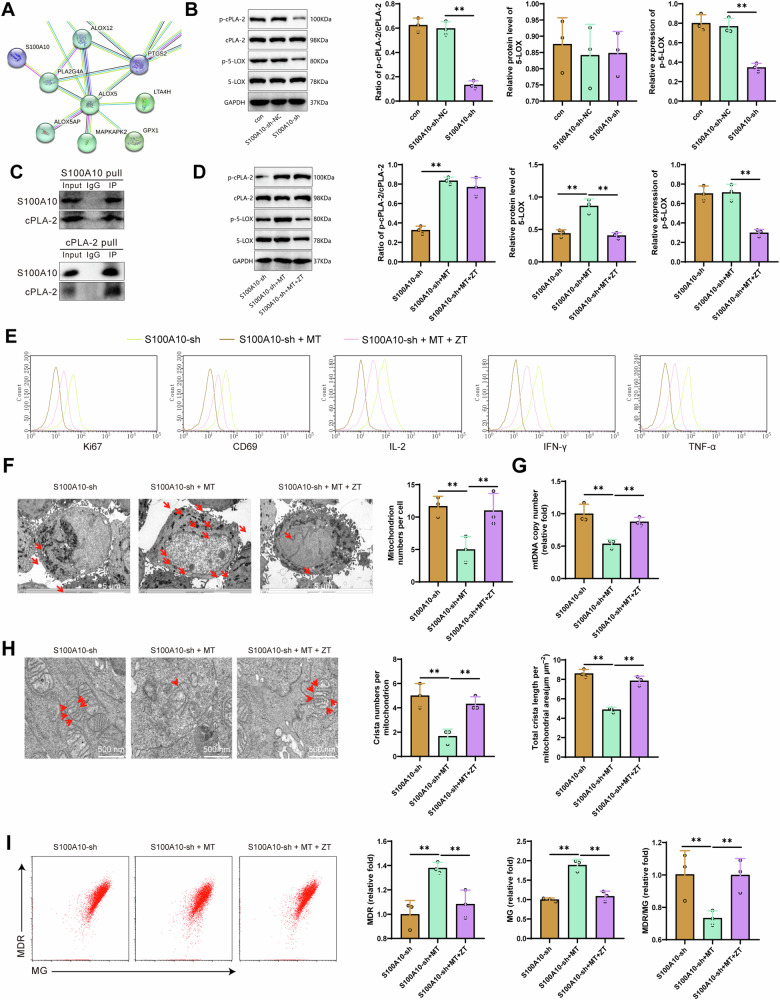
The impact of cPLA2 and 5-LOX on CD8^+^ T cell exhaustion. **A** PPI protein interaction network of S100A10 constructed using String. **B** Western blot analysis of p-cPLA2, cPLA2, 5-LOX and p-5-LOX protein expression in different MHCC97-L cell groups. **C** Co-IP analysis of the relationship between S100A10 and cPLA2. **D** Western blot analysis of p-cPLA2 and cPLA2 protein expression in different MHCC97-L cell groups. **E** Flow cytometry analysis of the expression levels of Ki67, CD69, IL-2, IFN-γ, and TNF-α in CD8^+^ T cells from different groups. **F** Transmission electron microscopy observation of the number of mitochondria in CD8^+^ T cells from different groups, with red arrows indicating mitochondria (scale bar = 5 μm). **G** RT-qPCR analysis of mtRNA copy numbers in CD8^+^ T cells from different groups. **H** Transmission electron microscopy observation of the number and total length of mitochondrial cristae in CD8^+^ T cells from different groups, with red arrows indicating mitochondrial cristae (scale bar = 500 nm). **I** Flow cytometry analysis of mitochondrial mass and membrane potential in CD8^+^ T cells from different groups. Cell experiments were repeated three times. * represents comparison between two groups, *p* < 0.05, *p* < 0.01, **p < 0.001.

To determine the specific molecular mechanism by which S100A10 induces CD8^+^ T cell exhaustion, we input the protein sequences encoded by differentially expressed genes related to lipid metabolism into the String website. We found a protein-protein interaction between S100A10 and PLA2G4A (Fig. 5A). Studies have shown that overexpression of PLA2G4A (cPLA2) could enhance lipid metabolism enzyme activity, accelerate lipid metabolism, and promote cancer cell proliferation [53–55]. Furthermore, based on existing research, it has been confirmed that ALOX5 (5-LOX) is a downstream factor of PLA2G4A (cPLA2) [56, 57]. We speculate that S100A10 may induce CD8^+^ T cell exhaustion through the cPLA2 and 5-LOX axis.

In in vitro experiments, we observed a significant reduction in the phosphorylation levels of cPLA2 protein in MHCC97-L cells transfected with S100A10-sh plasmid. However, there was no significant change in the protein content of 5-LOX, although the phosphorylation levels of 5-LOX protein showed a significant decrease (Fig. 5B). Previous studies have confirmed that phosphorylation of cPLA2 facilitates the release of arachidonic acid (AA), which is then metabolized into leukotrienes and lipoxins by the action of 5-LOX [56]. Our research suggests that S100A10 may be involved in this process. In addition, the Co-IP results showed a binding relationship between S100A10 protein and cPLA2 protein, suggesting that S100A10 protein may bind to cPLA2 protein and facilitate its phosphorylation (Fig. 5C).

Zileuton is a 5-lipoxygenase (5-LOX) inhibitor with a benzothiophene N-hydroxyurea structure that alleviates allergic and inflammatory states by inhibiting the biosynthesis of leukotrienes. Zileuton exerts its inhibitory effect on 5-LOX activity by coordinating with the iron ion at the active site and also exhibits weak reducing properties [58]. Additionally, studies have shown that post-treatment with Zileuton can suppress the levels of 5-LOX protein [59, 60]. Based on this, we separately used the cPLA2 activator Melittin or combined it with the 5-LOX inhibitor Zileuton to further explore the possible molecular mechanisms of S100A10-induced CD8^+^ T cell exhaustion in MHCC97-L cells transfected with S100A10-sh plasmid. Western blot results showed that compared to the S100A10-sh group, the cPLA2 protein phosphorylation level was significantly increased in the S100A10-sh+MT group. Compared to the S100A10-sh+MT group, using Zileuton did not affect the phosphorylation level of cPLA2. Compared to the S100A10-sh group, the S100A10-sh+MT group showed a significant increase in p-5-LOX expression, attributable to Melittin-induced cPLA2 phosphorylation. In contrast, compared to the S100A10-sh+MT group, the S100A10-sh+MT + ZT group exhibited a significant decrease in p-5-LOX expression, which was attributed to Zileuton’s inhibition of 5-LOX. Additionally, the use of Zileuton significantly reduced the expression of 5-LOX compared to the S100A10-sh+MT group (Fig. 5D). Flow cytometry analysis showed that compared with the S100A10-sh group, the proliferative capacity (Ki67) and cell vitality (CD69) of CD8^+^ T cells were reduced in the S100A10-sh+MT group, accompanied by decreased levels of IFN-γ, IL-2, and TNF-α, as well as a decrease in mitochondrial membrane potential. Compared with the S100A10-sh+MT group, the use of Zileuton enhanced the proliferative capacity (Ki67) and cell vitality (CD69) of CD8^+^ T cells, upregulated the levels of IFN-γ, IL-2, and TNF-α, and inhibited the decrease in mitochondrial membrane potential (Fig. 5E, I).

Furthermore, transmission electron microscopy results revealed that compared to the S100A10-sh group, the S100A10-sh+MT group exhibited a decrease in the number of mitochondria in CD8^+^ T cells, as well as a reduction in mtRNA copy numbers, mitochondrial cristae quantity, and length. In contrast, when comparing the S100A10-sh+MT group to the group treated with Zileuton, a decrease in mitochondrial quantity was observed in CD8^+^ T cells, while there was an upregulation in mtRNA copy numbers, mitochondrial cristae quantity, and length (Fig. 5F-H).

### LTB4 is the effector molecule that induces CD8^+^ T cell exhaustion by S100A10

Previous studies have shown that the upregulation of S100A10 in HCC cells may lead to an increase in the levels of leukotriene B4 (LTB4), which has been proven to be a pro-inflammatory factor [56]. Therefore, we believe that sustained stimulation with high concentrations of LTB4 may be one of the key factors leading to CD8^+^ T cell exhaustion.

**Fig. 6:**
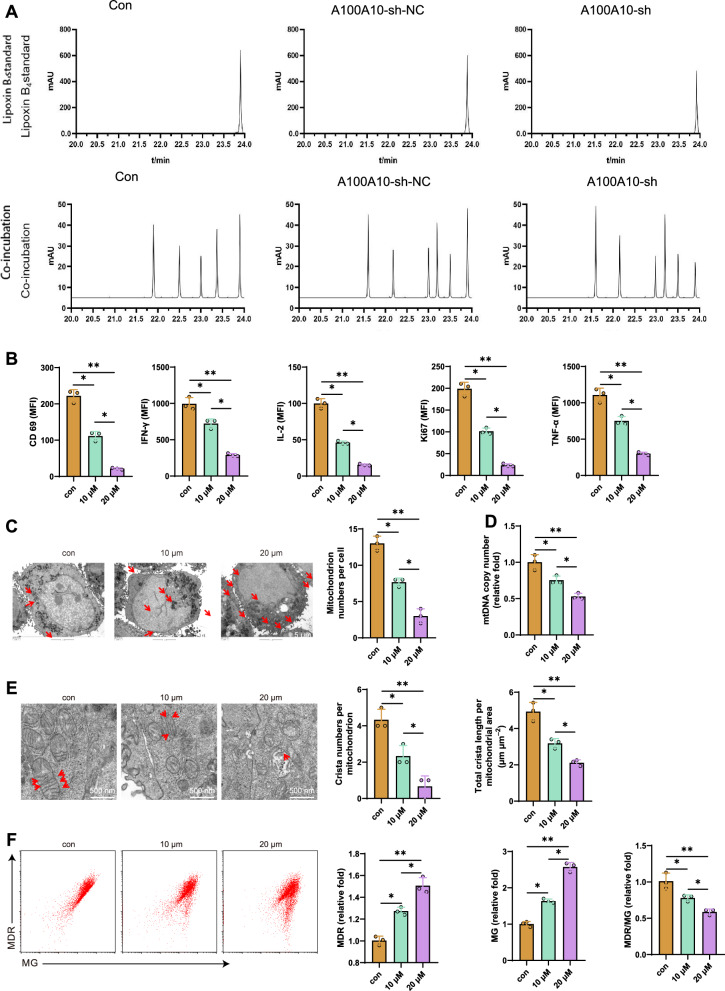
The effect of LTB4 on CD8^+^ T cells. **A** HPLC analysis of extracellular LTB4 levels in MHCC97-L cells. **B** Flow cytometry analysis of the expression levels of Ki67, CD69, IL-2, IFN-γ, and TNF-α in CD8^+^ T cells from different groups. **C** Transmission electron microscopy observation of the number of mitochondria in CD8^+^ T cells from different groups, with red arrows indicating mitochondria (scale bar = 5 μm). **D** RT-qPCR analysis of mtRNA copy numbers in CD8^+^ T cells from different groups. **E** Transmission electron microscopy observation of the number and total length of mitochondrial cristae in CD8^+^ T cells from different groups, with red arrows indicating mitochondrial cristae (scale bar = 500 nm). **F** Flow cytometry analysis of mitochondrial mass and membrane potential in CD8^+^ T cells from different groups. Cell experiments were repeated three times. * represents comparison between two groups, *p* < 0.05, *p* < 0.01, **p < 0.001.

We first measured the extracellular LTB4 levels in co-cultured MHCC97-L cells to test this hypothesis. HPLC results showed that the production of LTB4 was detectable in the extracellular matrix of MHCC97-L cells, while the content of extracellular LTB4 in MHCC97-L cells with silenced S100A10 was significantly decreased (Fig. 6A). Subsequently, we co-cultured CD8^+^ T cells with exogenous LTB4 for 48 h and analyzed the status of the CD8^+^ T cells post-culture. The results revealed that as the concentration of LTB4 increased, the proliferation capacity (Ki67) and cell viability (CD69) of CD8^+^ T cells decreased. Levels of IFN-γ, IL-2, and TNF-α production also decreased, along with a reduction in mitochondrial quantity, mtRNA copy numbers, mitochondrial cristae quantity, total length, and membrane potential. Furthermore, these effects demonstrated a dose-dependent relationship (Fig. 6B-F).

Furthermore, we investigated the status of CD8^+^ T cells isolated from HCC patients’ HCC tissues when co-cultured with exogenous LTB4. The results indicated that with increasing LTB4 concentration, the proliferation capacity (Ki67) and cell viability (CD69) of CD8^+^ T cells decreased. Levels of IFN-γ, IL-2, and TNF-α production were reduced, while mitochondrial quantity increased. Moreover, mtRNA copy numbers, mitochondrial cristae quantity, total length, and membrane potential all decreased, displaying a dose-dependent pattern (Figure S3).

To eliminate leukotrienes produced by HCC cells, we transfected MHCC97-L cells with S100A10 NC and S100A10-sh plasmids, compared them with untreated MHCC97-L cells, and co-cultured them with CD8^+^ T cells. Flow cytometry was employed to assess the CD8^+^ T cell status and validate the effector molecules of CD8^+^ T cell depletion. The results showed that co-culturing CD8^+^ T cells with MHCC97-L cells transfected with S100A10-sh plasmids enhanced the proliferation capacity (Ki67) and cell viability (CD69) of the CD8^+^ T cells. Additionally, there was an increase in the levels of IFN-γ, IL-2, and TNF-α production, while mitochondrial quantity decreased. Furthermore, mtRNA copy numbers, mitochondrial cristae quantity, total length, and membrane potential all increased (Figure S4).

### Depletion of S100A10 inhibits CD8^+^ T cell exhaustion in NOD-SCID mice

**Fig. 7:**
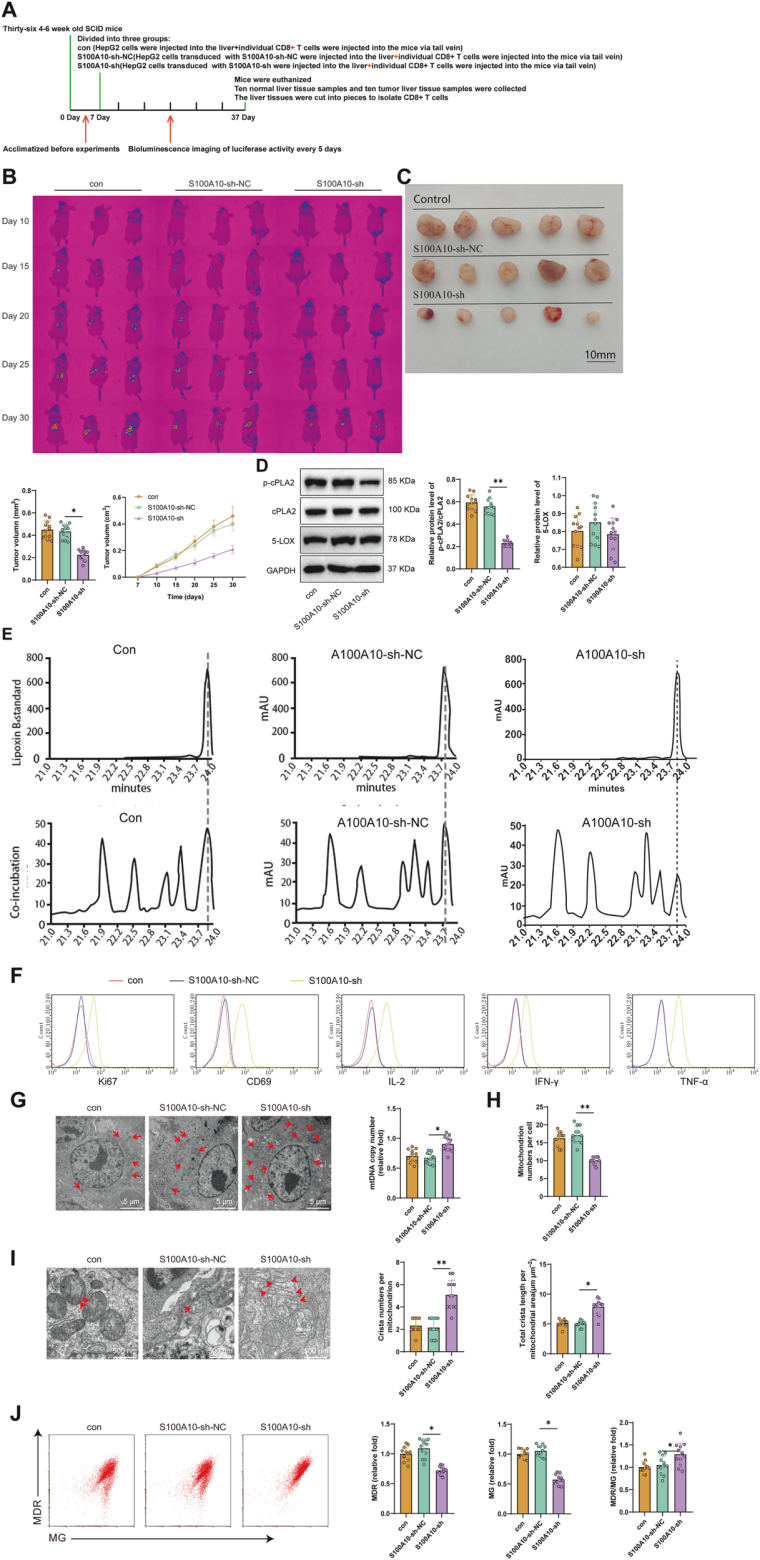
The impact of S100A10 silencing on NOD-SCID mice. **A** Construction of the NOD-SCID mouse model. **B** In vivo fluorescence imaging of tumor distribution in different groups of NOD-SCID mice. **C** Tumor volume and tumor growth rate in different groups of NOD-SCID mice after 30 days. **D** Western blot analysis of p-cPLA2, cPLA2, and 5-LOX protein expression in different tumor tissues. **E** HPLC analysis of LTB4 levels in different tumor tissues. **F** Flow cytometry analysis of Ki67, CD69, IL-2, IFN-γ, and TNF-α expression in tumor tissues. **G** Transmission electron microscopy observation of the number of mitochondria in CD8^+^ T cells in tumor tissues, with red arrows indicating mitochondria (scale bar = 5 μm). **H** RT-qPCR analysis of mtRNA copy numbers. **I** Transmission electron microscopy observation of the number and total length of mitochondrial cristae in CD8^+^ T cells in tumor tissues, with red arrows indicating mitochondrial cristae (scale bar=500 nm). **J** Flow cytometry analysis of mitochondrial mass and membrane potential in CD8^+^ T cells in tumor tissues. Each group consisted of 12 NOD-SCID mice. * represents comparison between two groups, *p* < 0.05, **p < 0.001.

To further investigate the impact of S100A10 on HCC in vivo, we injected MHCC97-L cells that were either not transfected, transfected with S100A10-sh-NC, or transfected with S100A10-sh plasmid into NOD-SCID mice to establish an HCC animal model (Fig. 7A). The results indicate that compared to the S100A10-sh-NC group, the tumor decreased in size, and the growth rate of MHCC97-L cells within the NOD-SCID mice decreased in the S100A10-sh group (Fig. 7B-C).

Through in vitro experiments, it is known that S100A10 induces CD8^+^ T cell exhaustion through the cPLA2 and 5-LOX pathway. We conducted tests on the obtained tumor tissues to further verify this conclusion. The Western blot results showed that compared to the S100A10-sh-NC group, the phosphorylation level of cPLA2 protein in the tumor tissue of the S100A10-sh group was significantly reduced, but there was no significant change in the protein content of 5-LOX (Fig. 7D).

In addition, we measured the levels of LTB4 in tumor tissues from various groups. HPLC results showed that compared with the S100A10-sh-NC group, the content of LTB4 in tumor tissues was significantly decreased in the S100A10-sh group (Fig. 7E).

Meanwhile, the flow cytometry results showed that compared to the S100A10-sh-NC group, the proliferation capacity (Ki67) and cell viability (CD69) of CD8^+^ T cells in tumor tissue were enhanced in the S100A10-sh group. Additionally, the IFN-γ, IL-2, and TNF-α levels were upregulated, and the mitochondrial membrane potential increased (Fig. 7F, J). Transmission electron microscopy results revealed that in comparison to the S100A10-sh-NC group, the S100A10-sh group showed an increase in the number of mitochondria in CD8^+^ T cells, as well as an increase in mtRNA copy number, the quantity and length of mitochondrial cristae (Fig. 7G-I).

Furthermore, for the construction of an HCC model, we selected immunocompetent BALB/c mice to investigate the impact of S100A10 silencing on CD8^+^ T cell exhaustion. The results from flow cytometry revealed that compared to the S100A10-sh-NC group, in the S100A10-sh group, the proliferative capacity (Ki67) and cell vitality (CD69) of CD8^+^ T cells in the tumor tissue increased. Additionally, the levels of IFN-γ, IL-2, and TNF-α were upregulated, and mitochondrial membrane potential increased (Figure S5).

## Discussion

HCC stands as one of the most formidable malignancies globally, accounting for a substantial proportion of cancer-related mortalities [61]. Its intricate etiology is woven with multiple factors, notably chronic viral infections, alcoholic liver diseases, and metabolic syndromes [62]. Despite advances in diagnostic modalities and therapeutic regimens, the prognosis for HCC remains disconcertingly bleak, primarily due to factors like late-stage diagnosis and the tumor’s adeptness at evading the host’s immune system [63]. Immune evasion, a pivotal player in HCC progression, undermines the effectiveness of immunotherapeutic approaches and demands rigorous scientific exploration [8]. Delving into the molecular actors, like S100A10, which might drive these immune escape mechanisms, becomes imperative to unveil potential therapeutic targets and improve clinical outcomes for HCC patients.

In our study, we observed in a nude mouse xenograft tumor model that, compared to the Normal group, the Tumor group exhibited a significant increase in the expression of exhaustion markers such as PD-1 and TIM-3 on CD8^+^ T cells within the tumor tissues. Additionally, levels of cytokines like IL-2 and IFN-γ decreased, along with decreased cell proliferation and vitality and noticeable mitochondrial impairment. These findings suggest the occurrence of functional exhaustion in CD8^+^ T cells induced by HCC. T cell exhaustion, a known mechanism widely described to inhibit CD8^+^ T cell proliferation and their capability to kill tumor cells, has been linked to several genes highly expressed in various tumor tissues like CEACAM1 and SIRT7 [64–67]. Furthermore, the hallmark of exhausted CD8^+^ T cells is the progressive loss of effector functions, strong activation of the T cell exhaustion driver TOX, and continuous expression of inhibitory receptors such as PD-1, LAG-3, TIM-3, and CTLA-4 [68, 69]. Our research reveals the presence of CD8^+^ T cell exhaustion within HCC tissues.

Utilizing RNA-seq technology and bioinformatics analysis, our study revealed that S100A10 is highly expressed in tumor tissues, which can predict the survival time of HCC patients and is strongly correlated with the infiltration level of CD8^+^ T cells in HCC tissues. The protein-protein interaction (PPI) network illustrated that S100A10 interacts with the protein cPLA-2 encoded by the lipid metabolism-related gene PLA2G4A, suggesting a potential link between S100A10 and HCC-induced exhaustion of CD8^+^ T cells through lipid metabolism reprogramming. As a member of the S100 protein family, S100 calcium-binding protein A10 (S100A10) regulates various biological functions, such as cell motility, phosphorylation, and signal transduction pathways [70]. The cPLA2 and 5-LOX pathway plays a crucial role in lipid metabolism and has a significant impact on reprogramming-induced CD8^+^ T cell exhaustion [71]. Investigating the influence of the cPLA2 and 5-LOX pathway on CD8^+^ T cell exhaustion can aid in the development of more effective therapeutic strategies for HCC. Early studies identified the oncogenic role of S100A10 in ovarian, gastric, breast, and renal cell cancers [72, 73]. Recent research indicates that S100A10 serves as a novel predictive factor for clinical diagnosis, prognosis, and immunotherapy response in lung cancer patients [74]. Our findings suggest that S100A10 could serve as a novel prognostic factor for HCC patients.

The primary recognized function of S100A10 is to regulate the conversion of plasminogen to plasmin by interacting with membrane protein A2. Plasmin, in conjunction with other proteases, induces the degradation of the extracellular matrix (ECM), a crucial step in tumor progression [75]. It has been reported that S100A10 interacts with cPLA2, inhibiting cPLA2 activity and A2 release. The released A2 can act as a second messenger, activating other apoptosis-related enzymes such as phospholipase, ultimately leading to the exhaustion of CD8^+^ T cells [76]. Previous literature has linked the proliferation and stemness potential of hepatocellular carcinoma (HCC) cells to the levels of 5-LOX. Elevated 5-LOX levels enhance HCC cell proliferation and stemness potential, while inhibition of 5-LOX activity regulates HCC progression [16]. In our study, we reveal that S100A10 initiates lipid metabolism reprogramming and upregulates LTB4 levels by activating the cPLA2 and 5-LOX axis. This cascade promotes CD8^+^ T cell exhaustion in HCC tissues, enabling the immune escape of HCC cells and consequently impacting the growth and migration of HCC cells.

In our in vitro experiments, we discovered that the silencing of S100A10 can inhibit the exhaustion of CD8^+^ T cells induced by co-culturing with MHCC97-L cells, simultaneously suppressing the cell viability of MHCC97-L cells. This effect is associated with cPLA2 and 5-LOX in a manner that suggests S100A10 can induce CD8^+^ T cell exhaustion by initiating lipid metabolism reprogramming via the cPLA2 and 5-LOX axis. Extensive research indicates that cPLA2 is a crucial enzyme in membrane phospholipid metabolism, unsaturated fatty acid metabolism, and PGE2 synthesis; its abnormal expression can lead to the onset of various diseases [77–81]. Moreover, studies demonstrate a close association between lipid metabolism reprogramming and cancer progression, where lipid accumulation is considered a significant hallmark of cancer cell invasiveness. This suggests that lipid metabolism reprogramming may occur during cancer development [82, 83]. Furthermore, multiple studies indicate that lipid accumulation in tumor tissues can mediate the exhaustion of infiltrating T cells in the tumor microenvironment [84–86]. Our research unequivocally demonstrates that S100A10 can induce CD8^+^ T cell exhaustion through lipid metabolism reprogramming, with its mechanism of action linked to the cPLA2 and 5-LOX axis.

Through in vivo experiments, we discovered that silencing S100A10 inhibited the growth and migration of tumors in NOD-SCID mice and restored the functionality of CD8^+^ T cells within the tumor tissues, indicating effective suppression of CD8^+^ T cell exhaustion in tumor tissues by S100A10 silencing, thereby inhibiting immune escape of HCC cells. Previous research indicates the critical role of T cell exhaustion in immune dysfunction and tumor immune evasion [69]. Among all tumor-infiltrating lymphocytes, CD8^+^ T cells represent the primary subset conducting anti-tumor immunity by executing T cell receptor-mediated killing of malignant cells. The correlation between CD8^+^ T cell infiltration and improved overall survival rates in cancer patients has been well established [87, 88]. Our findings suggest that S100A10 holds promise as a novel therapeutic target for HCC.

**Fig. 8:**
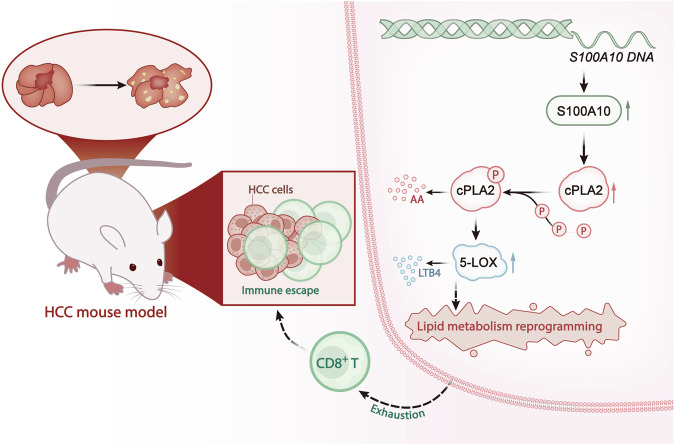
Molecular mechanism of S100A10 inducing CD8^+^ T cell exhaustion and promoting HCC cell immune escape via cPLA2 and 5-LOX axis.

In conclusion, we preliminarily propose the following hypothesis: S100A10 may activate the cPLA2 and 5-LOX-LOX axis, where signal activation initiates lipid metabolism reprogramming, upregulates LTB4 levels, thereby promoting CD8^+^ T cell exhaustion in HCC tissues, leading to immune escape of HCC cells, ultimately affecting the growth and migration of HCC cells (Fig. 8). Our research offers new perspectives and some theoretical basis for treating HCC. However, there are limitations as we did not assess the relevant products generated by S100A10-induced lipid metabolism reprogramming in MHCC97-L cells through metabolomics. Further validation in clinical samples is warranted.

However, every study has its limitations. In the present study, potential limitations may arise due to an inadequate sample size, which could impact the generalizability of the findings. Furthermore, the dataset chosen may exhibit bias and may not comprehensively depict the biological information traits of all patients with liver cancer. In the future, we anticipate the involvement of larger and more representative samples in research, along with a more thorough investigation of the downstream mechanisms associated with S100A10. This will provide a better understanding of the role and therapeutic potential of S100A10 in HCC.

### Supplementary information

Supplemental Materials

## Data Availability

The data that supports the findings of this study are available on request from the corresponding author.
