# Supplementary material for: Elucidating the role of S100A10 in CD8+ T cell exhaustion and HCC immune escape via the cPLA2 and 5-LOX axis
Source: Cell Death Dis. 2024 Aug 8;15(8):573. doi: 10.1038/s41419-024-06895-0 (PMC11310305; doi:10.1038/s41419-024-06895-0)
Supplement: Supplementary file 1 — Supplemental Materials [file 41419_2024_6895_MOESM1_ESM.pdf]

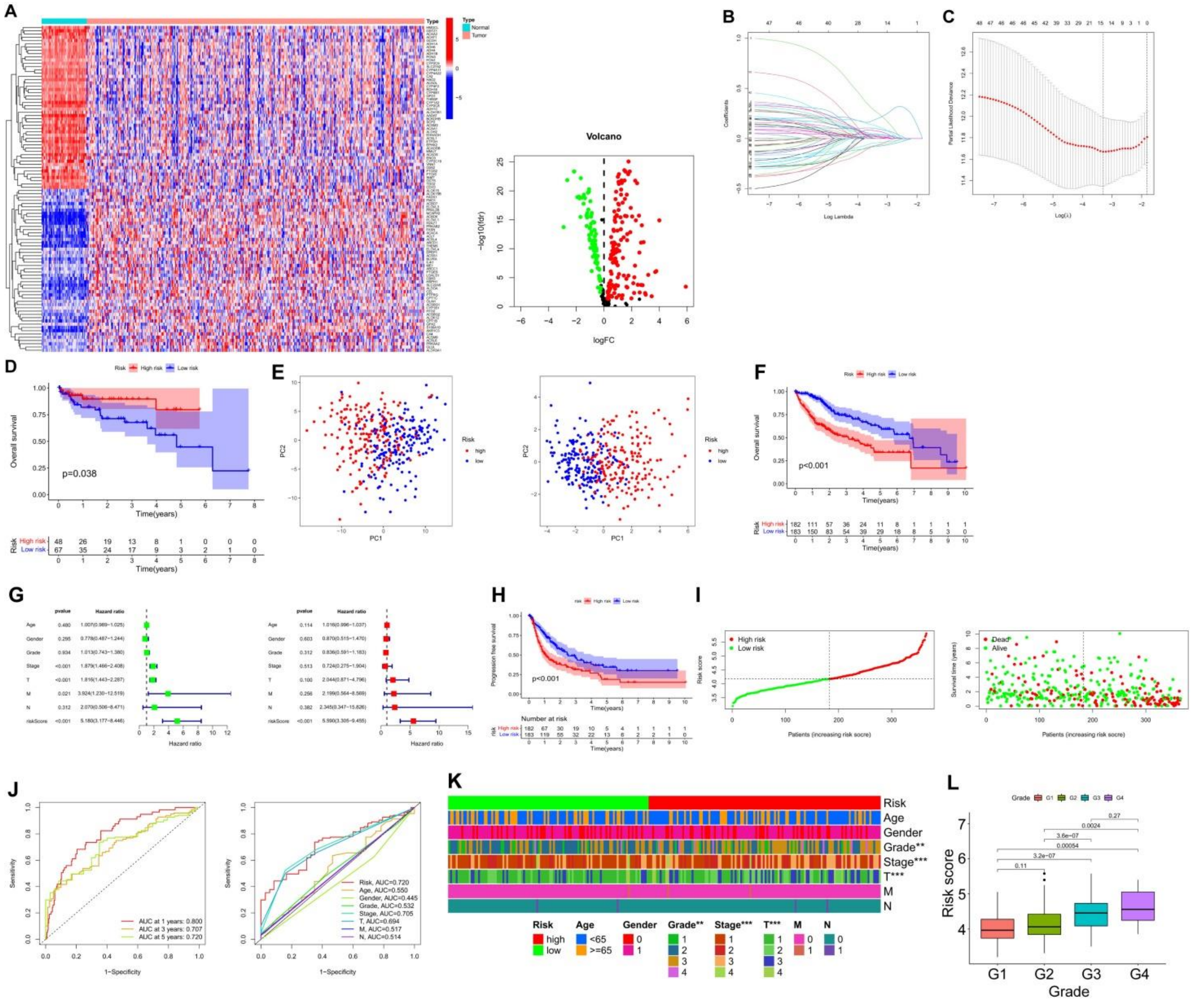

Figure S1 Construction and validation of the lasso regression model and accuracy evaluation for HCC patient prognosis.

Note: (A) Heatmap and volcano plot of TCGA lipid metabolism-related gene expression. Red represents upregulation, and green represents downregulation. (B) Change trajectories of all variables used in the Lasso regression model. (C) Many differentially expressed lipid metabolism-related genes were used in the Lasso model (15 genes shown in the figure). (D) The survival time difference between high-risk and low-risk groups in the GSE76427 dataset was analyzed using the model. (E) PCA clustering plots of lipid metabolism-related differentially expressed genes (left) and genes used in the model (right) in high-risk and low-risk groups of HCC patients from the TCGA database, with red representing the high-risk group and blue representing the low-risk group. (F) Overall survival analysis between high-risk and low-risk groups of HCC patients from the TCGA database. (G) Univariate (left) and multivariate (right) analyses of different classification methods for predicting HCC patient prognosis in the TCGA database. (H)

Progression-free survival plots between high-risk and low-risk groups of HCC patients from the TCGA database. (I) Risk curves and heatmaps between high-risk and low-risk groups of HCC patients from the TCGA database. (J) ROC curves for predicting the prognosis of HCC patients for 1-5 years based on risk values (left) and different clinical characteristics (right). (K) Correlation heatmap between clinical characteristics and high-risk and low-risk groups of HCC patients from TCGA database. (L) Analysis of differences in tumor grade between high-risk and low-risk groups of HCC patients from the TCGA database. * represents a comparison between two groups, *$p<0.05$.

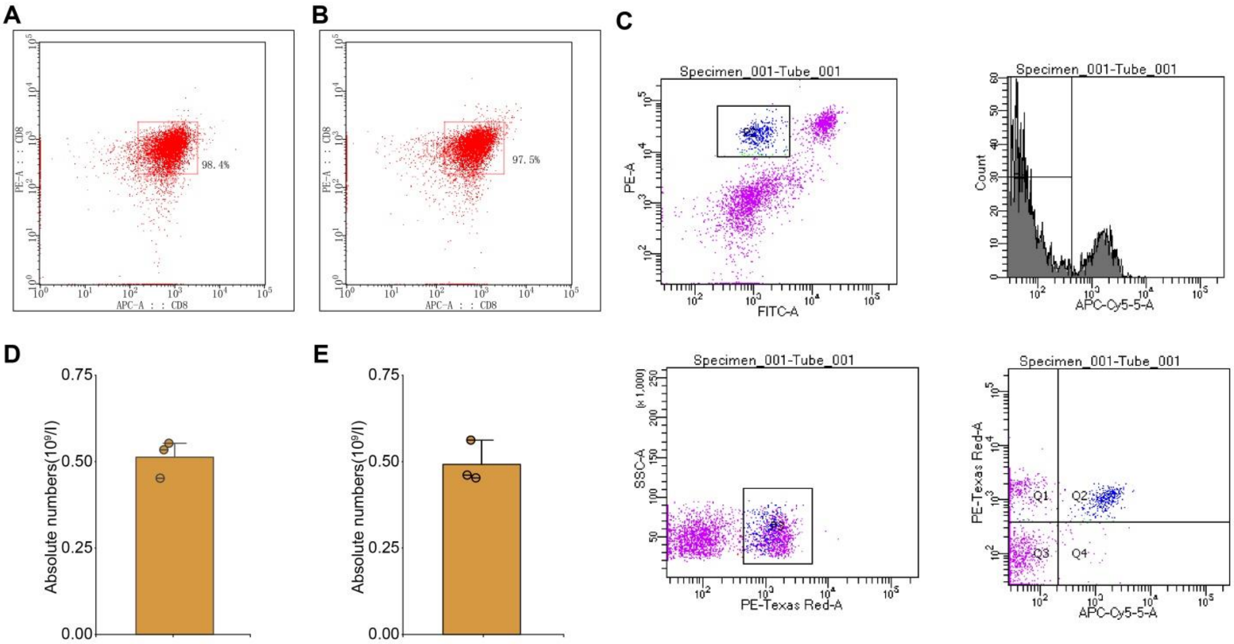

Figure S2 Detection of CD8+ T cell purity and absolute quantity.

Note: (A) Flow cytometry analysis of CD8+ T cell purity extracted from nude mice (n=10). (B) Flow cytometry analysis of CD8+ T cell purity extracted from human peripheral blood. (C) Gate strategy for CD8+ T cells extracted from nude mice. (D) Flow cytometry analysis of absolute CD8+ T cell counts extracted from nude mice (n=10). (E) Flow cytometry analysis of absolute CD8+ T cell counts extracted from human peripheral blood.

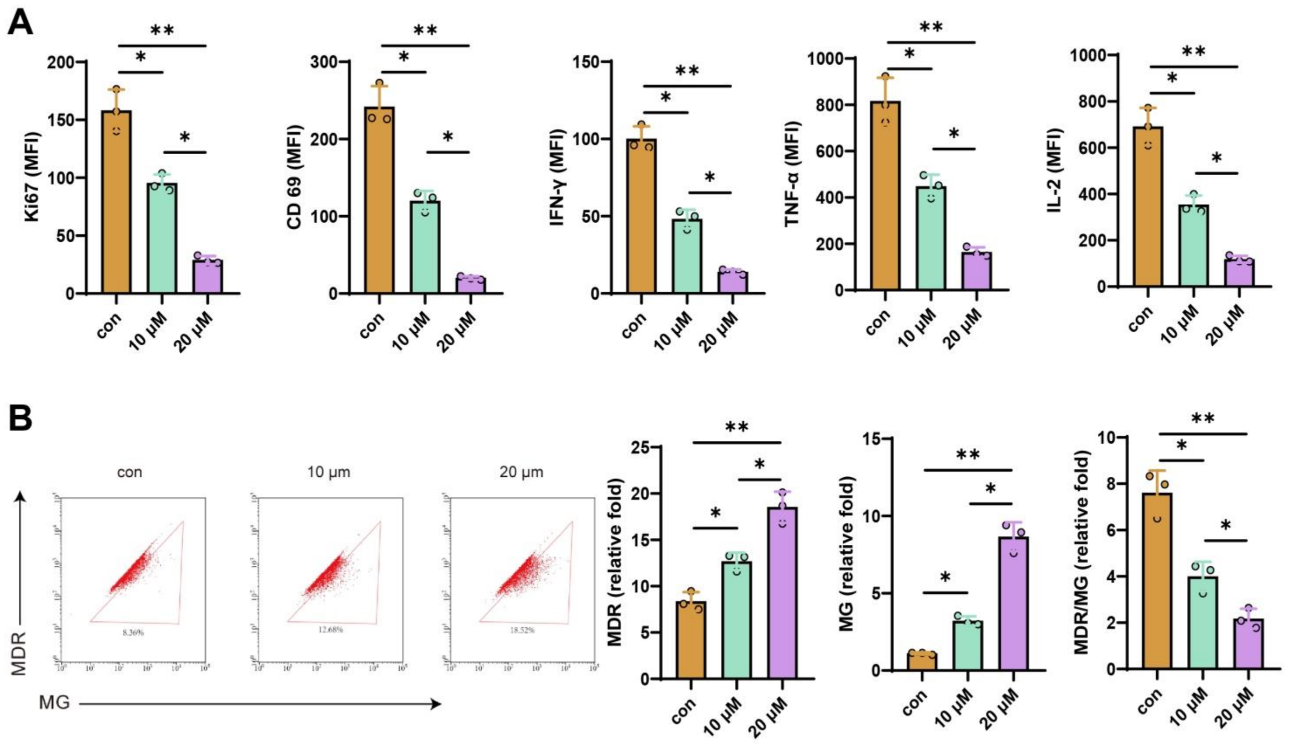

Figure S3. The impact of LTB4 on CD8+ T cells in HCC patients' HCC tissues.

Note: (A) flow cytometry was utilized to assess the expression levels of Ki67, CD69, IL-2, IFN-γ, and TNF-α in CD8+ T cells within each group. Additionally, (B) flow cytometry was employed to analyze the mitochondrial quality and membrane potential of CD8+ T cells in each group. The cell experiments were repeated three times, with * denoting comparisons between the two groups, $p < 0.05$, $p < 0.01$, and **$p < 0.001$.

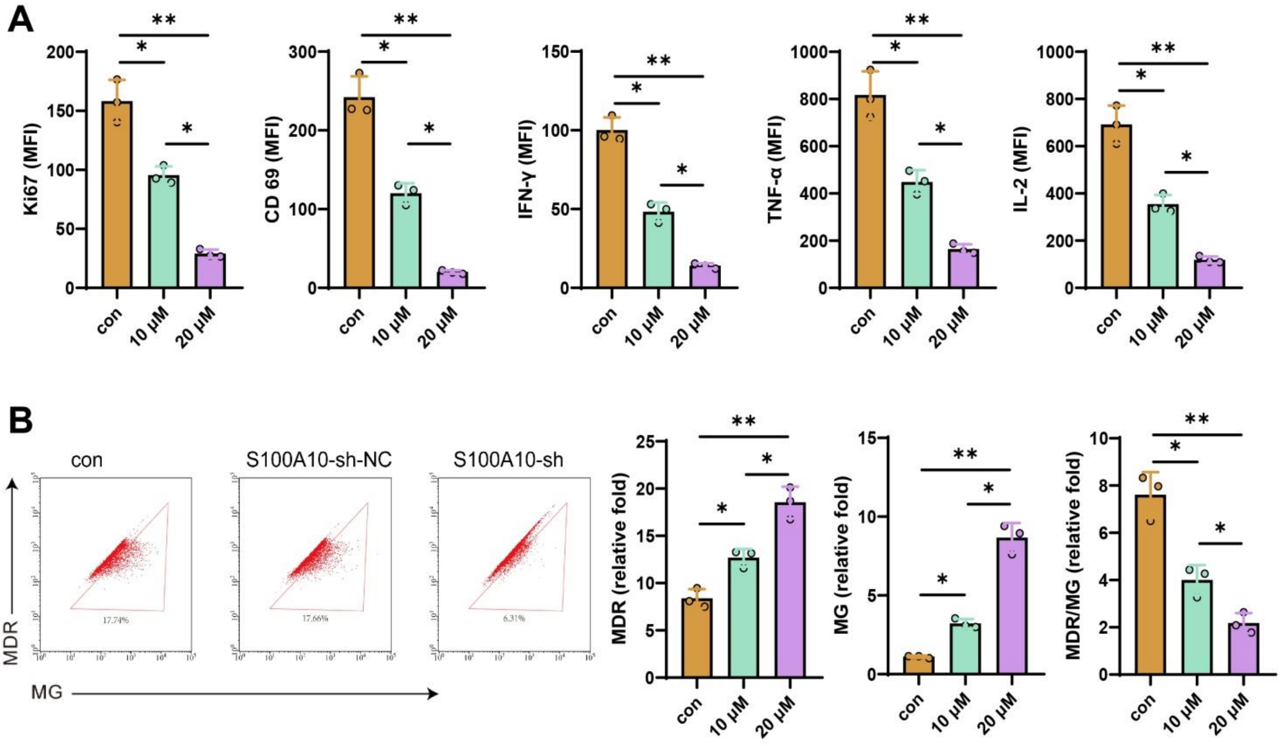

Figure S4. The Influence of S100A10-sh Plasmid Treatment on MHCC97-L Cells on CD8+ T Cells. Note: (A) Flow cytometry was used to assess the expression levels of Ki67, CD69, IL-2, IFN-γ, and TNF-α in CD8+ T cells in each group. (B) Flow cytometry was used to evaluate the mitochondrial quality and membrane potential of CD8+ T cells in each group. The cell experiments were conducted three times. * Indicates comparison between two groups, $p < 0.05$, $p < 0.01$, **$p < 0.001$.

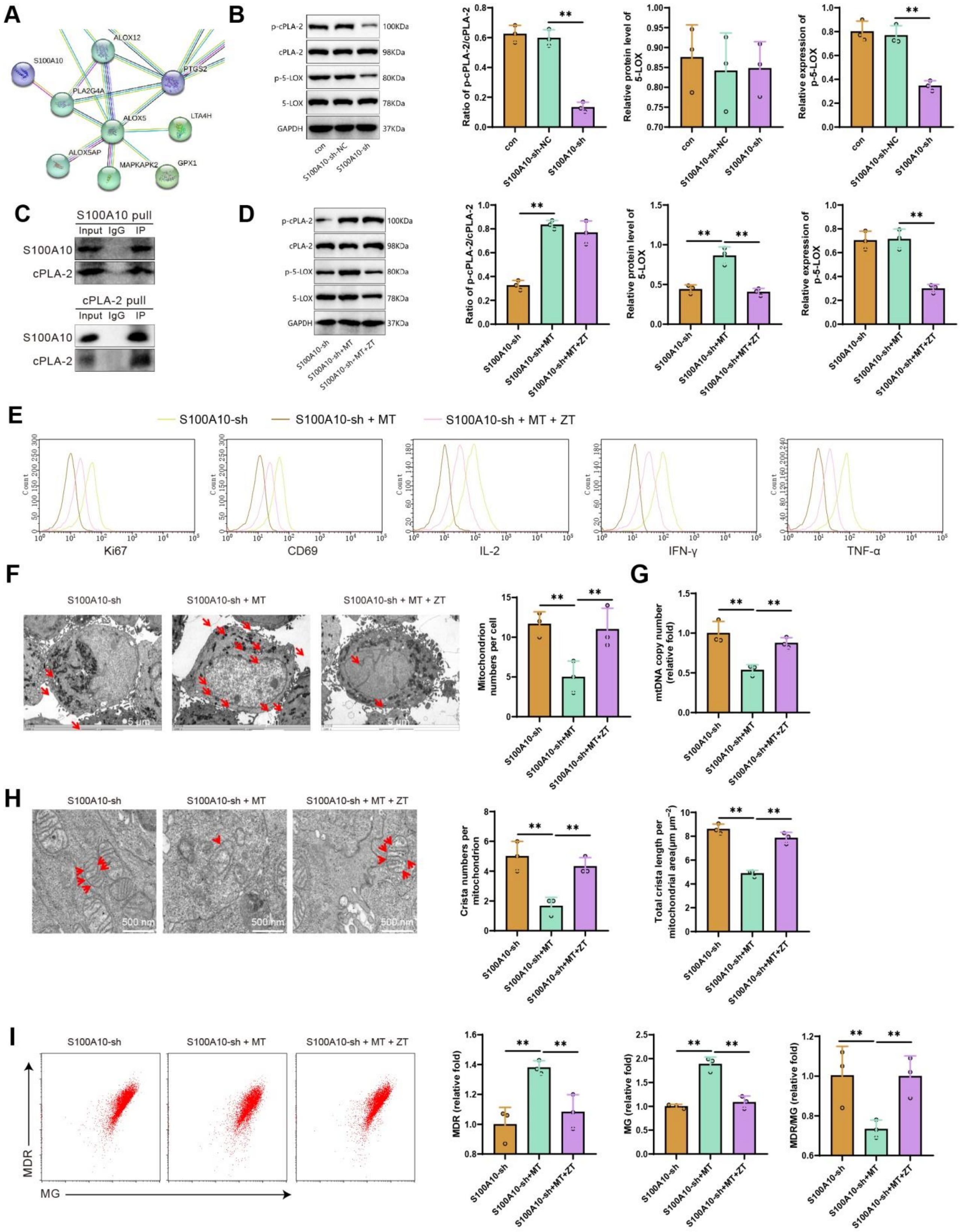

Figure S5. The Effect of Silenced S100A10 on BALB/c Mice Note: (A) Flow cytometry was used to assess the expression of Ki67, CD69, IL-2, IFN-γ, and TNF-α. (B) Flow cytometry was employed to evaluate the mitochondrial mass and membrane potential of CD8+ T cells in the tumor

tissues of each group. Each group consisted of 12 BALB/c mice, * indicates a comparison between the two groups,*p<0.05, **p<0.001.

Table S1. Human shRNA sequences

shRNA	Sequence (5'-3')
S100A10-sh-NC	CCATTGATGACTCATGCAATT
S100A10-sh-1	CCATTGCATGCAATGACTATT
S100A10-sh-2	CCATGATGTTTACATTTCACA

Table S2. Mouse shRNA sequences

shRNA	Sequence (5'-3')
S100A10-sh-NC	CCATTGATGACTCATGCAATT
S100A10-sh-1	CCAGAGCTTTCTATCACTAGT
S100A10-sh-2	CCAGAGCTTTCTATCACTAGT

Table S3. Primer sequences for RT-qPCR

Gene	Sequence
S100A10 (human)	Forward: 5'-TCAAAAAGACCCTCTGGCTGT-3'
	Reverse: 5'-AGCTCTGGAAGCCCACTTTG-3'
GAPDH (human)	Forward: 5'-GAAAGCCTGCCGGTGACTAA-3'
	Reverse: 5'-AGGAAAAGCATCACCCGGAG-3'

Note: S100A10: S100 calcium binding protein A10; GAPDH: glyceraldehyde-3-phosphate dehydrogenase

Table S4. Primer sequences for RT-qPCR

Gene	Sequence
S100A10 (mouse)	Forward: 5'- ATGCCATCCCAAATGGAGCA-3'
	Reverse: 5'- GAACTCCCGTTCCATGAGCA-3'
GAPDH (mouse)	Forward: 5'- GGGTCCCAGCTTAGGTTCAT-3'
	Reverse: 5'- CTCGTGGTTCACACCCATCA -3'

Note: S100A10: S100 calcium binding protein A10; GAPDH: glyceraldehyde-3-phosphate dehydrogenase

Table S5. Expression of 15 lipid metabolism-related differentially expressed genes used for the construction of the Lasso model in TCGA database

Gene	conMean	treatMean	logFC	*p* value	FDR
ACOT7	4.992132	8.89522861	0.833375602	2.19E-10	4.77E-10
APEX1	27.850782	57.69279679	1.050673367	1.56E-23	2.80E-22
PON1	192.227494	108.8454693	-0.820533341	2.99E-11	7.40E-11
S100A10	40.67761	155.164862	1.931495066	1.25E-18	8.68E-18
PRDX6	184.530402	240.5942912	0.382743886	0.000687505	0.000951929
CYP2C9	235.856068	98.23148316	-1.263649331	2.95E-17	1.70E-16
SMS	11.995218	22.81561979	0.927562468	3.92E-17	2.12E-16
ACSL3	7.342836	12.61280455	0.780479821	2.64E-09	5.23E-09
HCCS	6.023814	9.11926738	0.598240702	9.95E-11	2.28E-10
ACADS	76.637246	34.45515588	-1.153325833	1.71E-21	2.01E-20
PRKAA2	0.293544	1.72642754	2.55614111	2.10E-10	4.60E-10
ME1	2.802036	9.005369519	1.684309984	0.000353336	0.00050745
ACAT1	60.62131	35.22196257	-0.78334973	5.30E-16	2.26E-15
UGDH	23.903952	43.62564786	0.8679274	0.000133629	0.000201183
METAP1	4.308406	6.802695187	0.658952239	1.14E-10	2.57E-10

Full and uncropped western blots

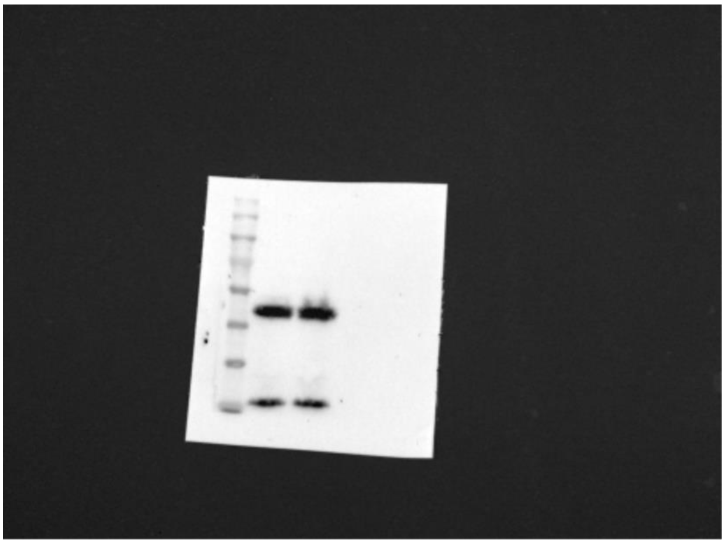

Figure 2D-1

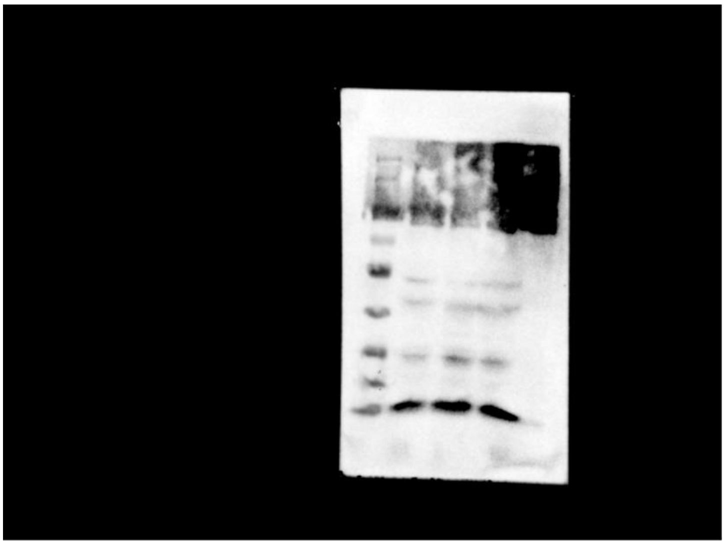

Figure 2D-2

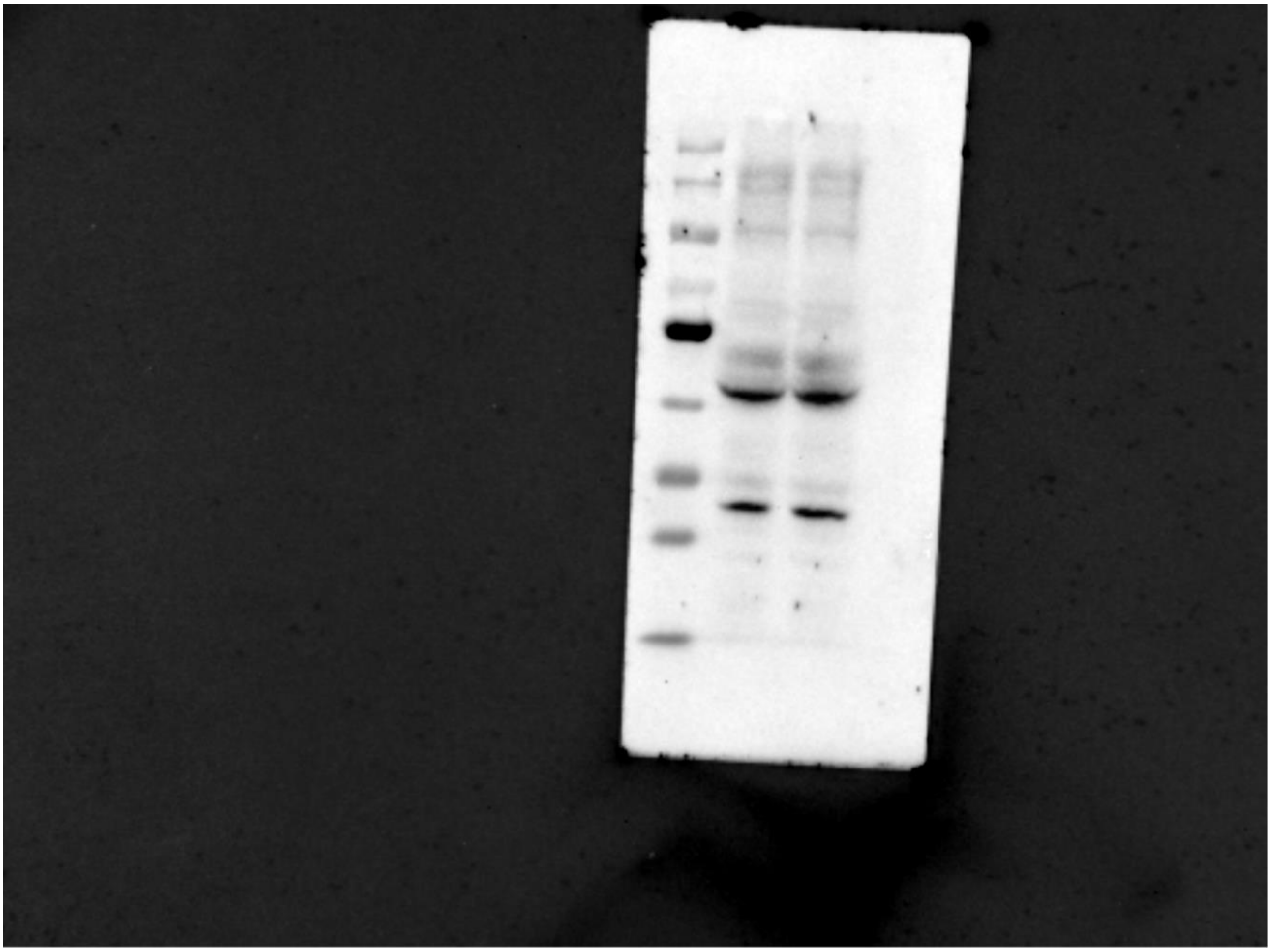

Figure 2D-3

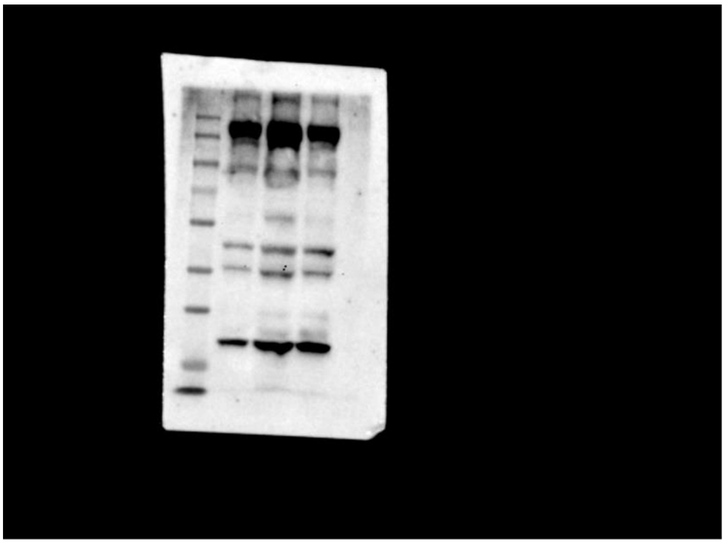

Figure 2D-4

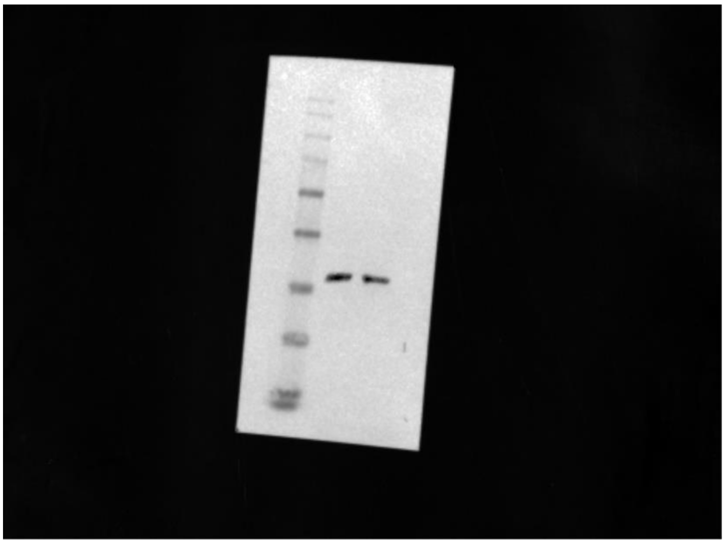

Figure 2D-5

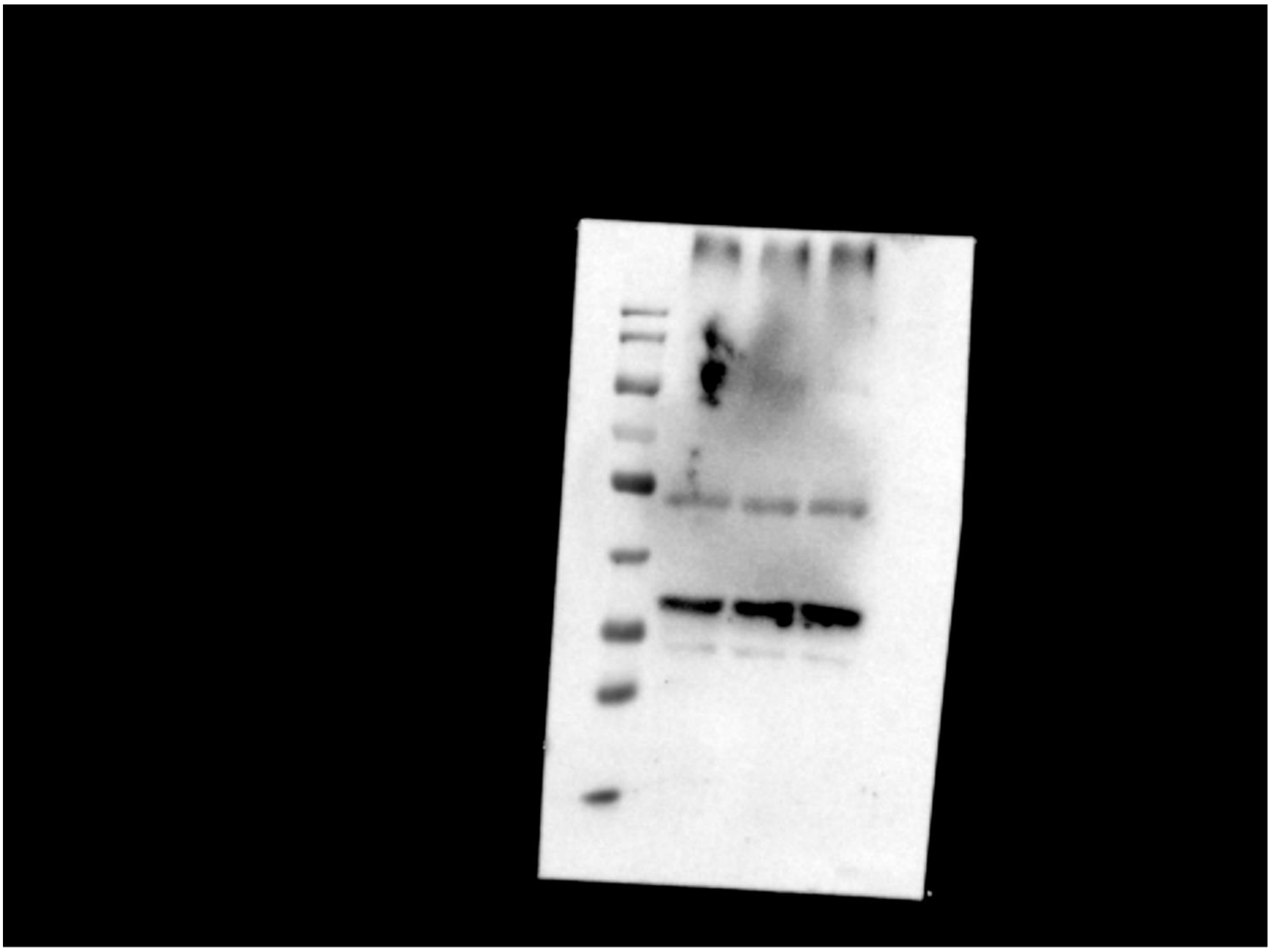

Figure 2D-6

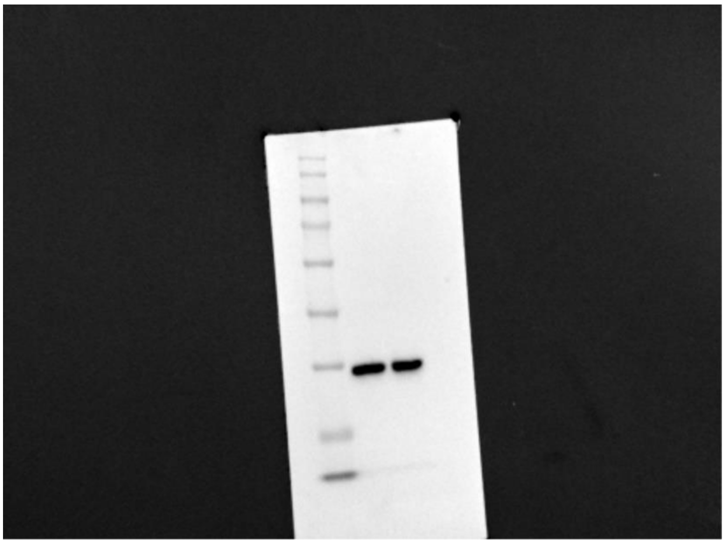

Figure 2D-7

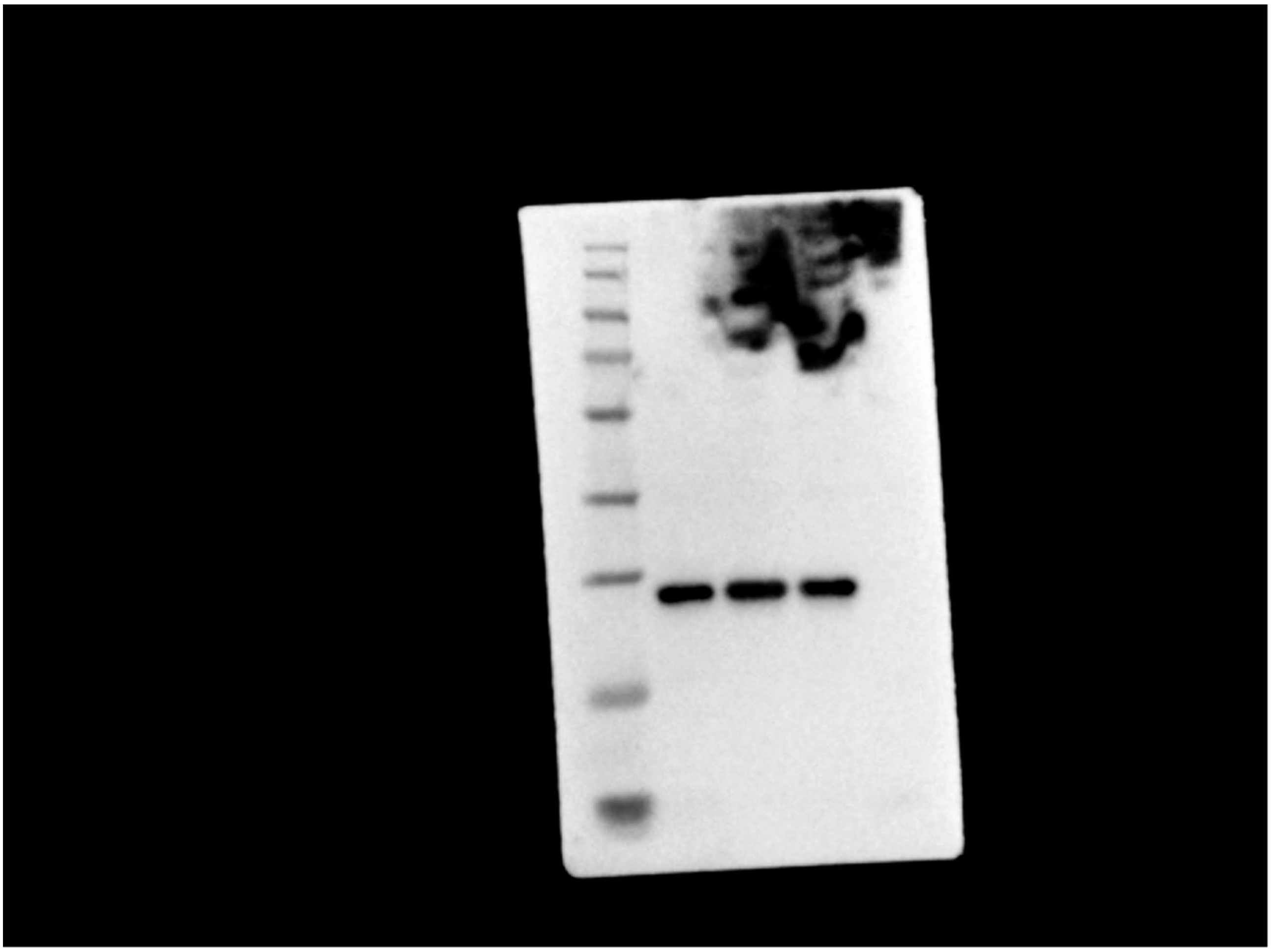

Figure 2D-8

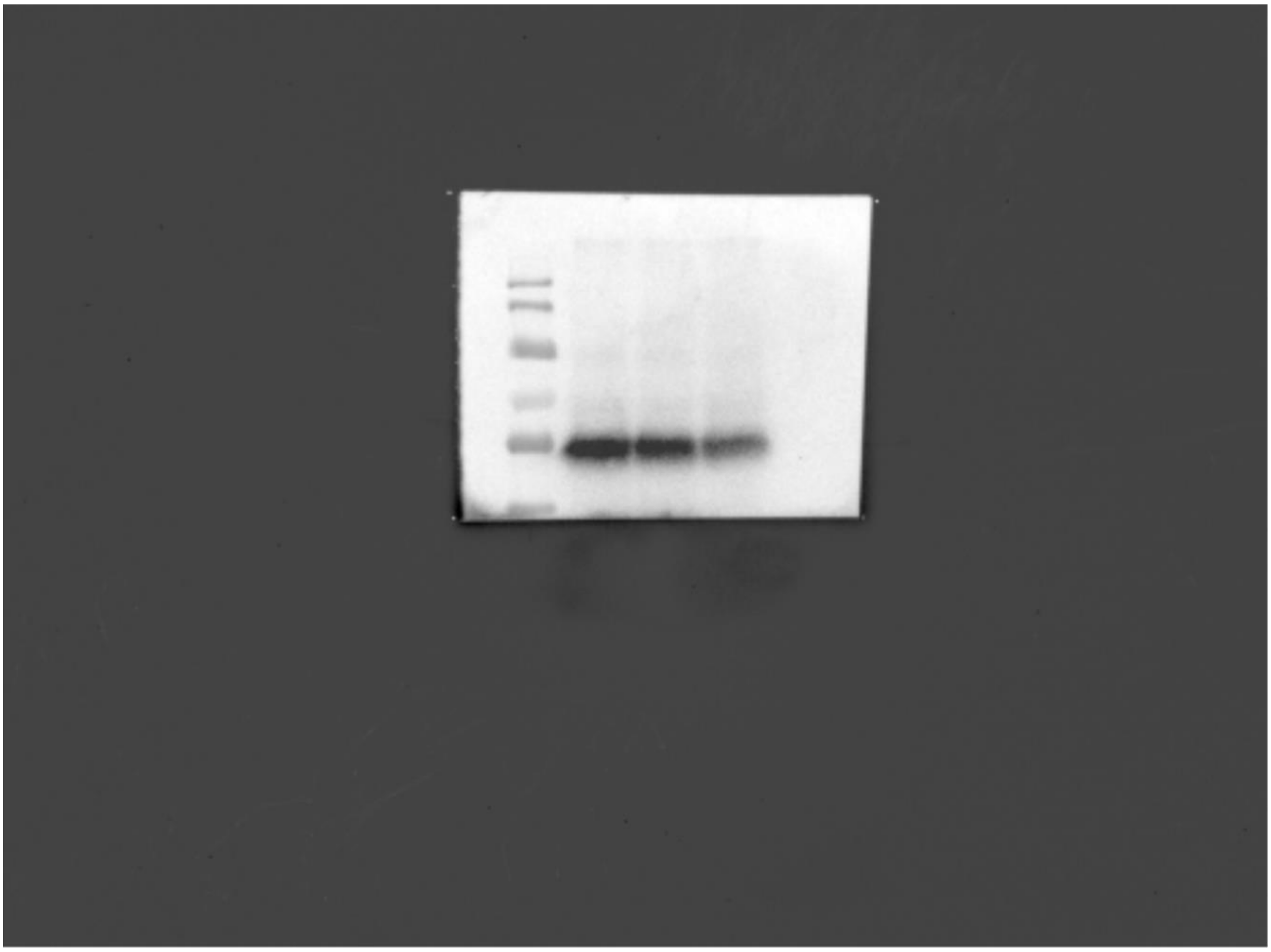

Figure 4C-1

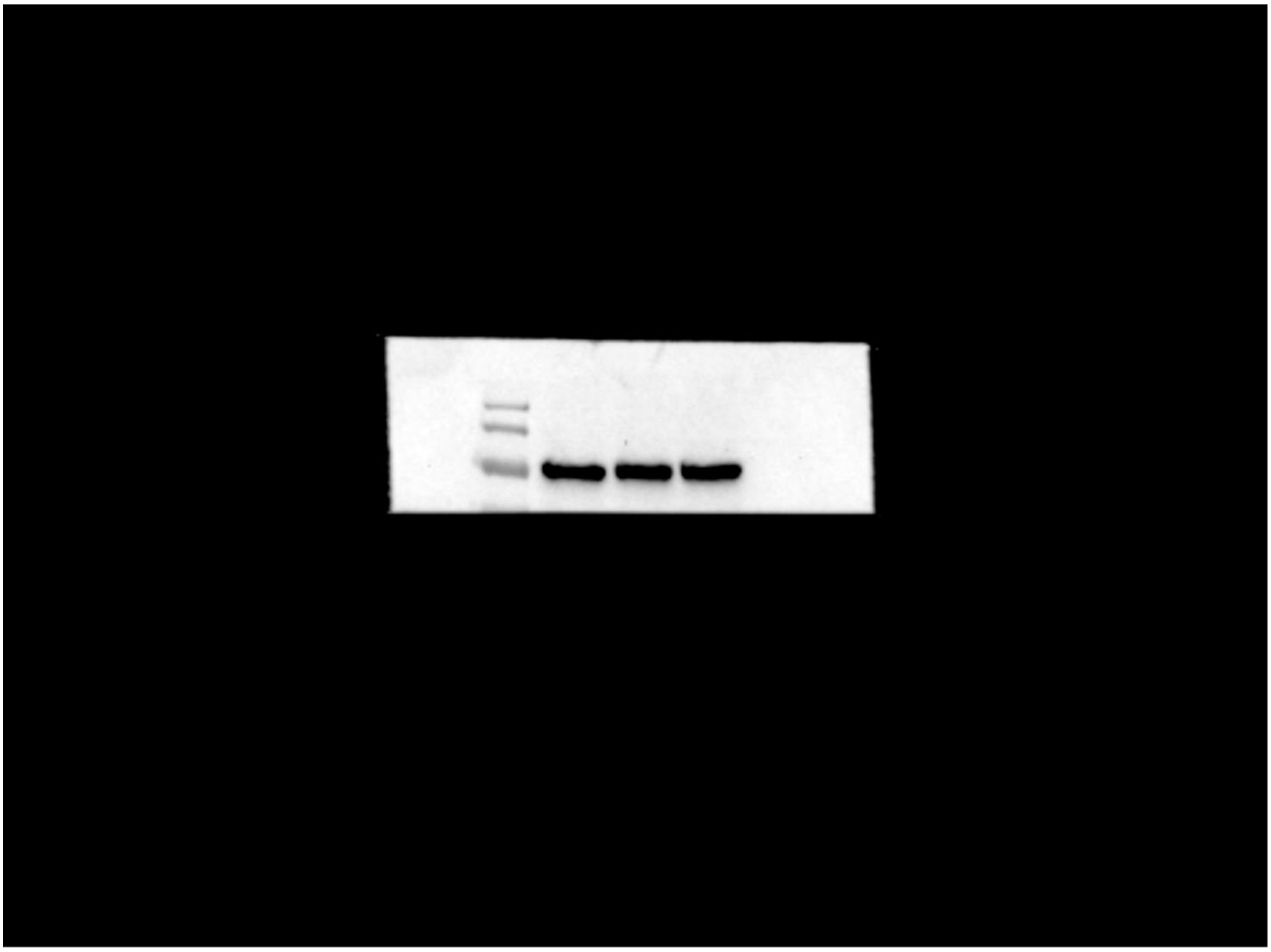

Figure 4C-2

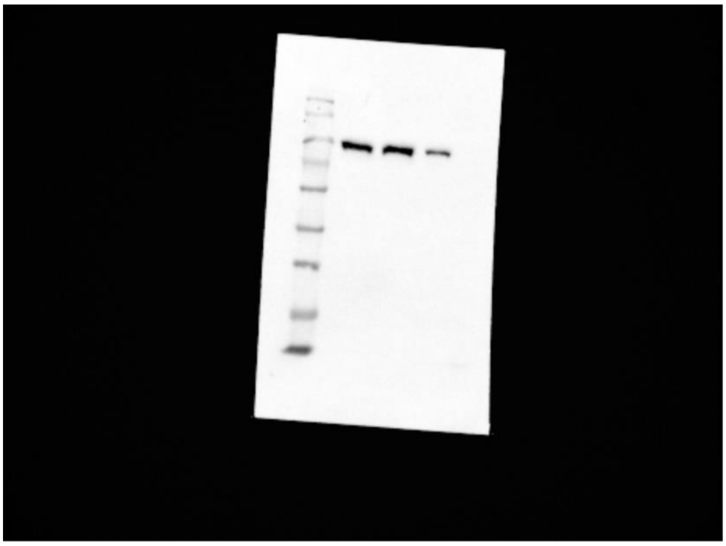

Figure 5B-1

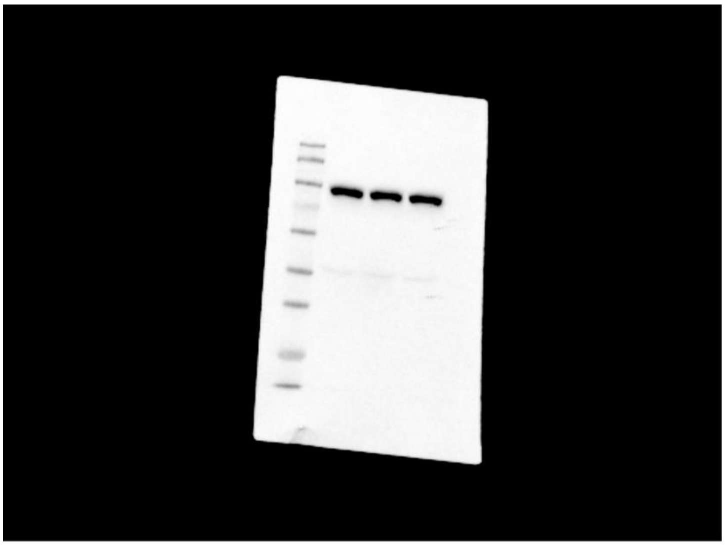

Figure 5B-2

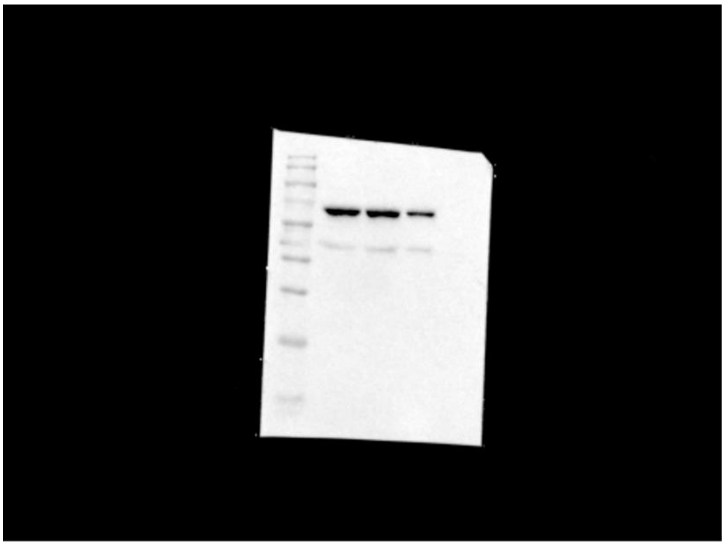

Figure 5B-3

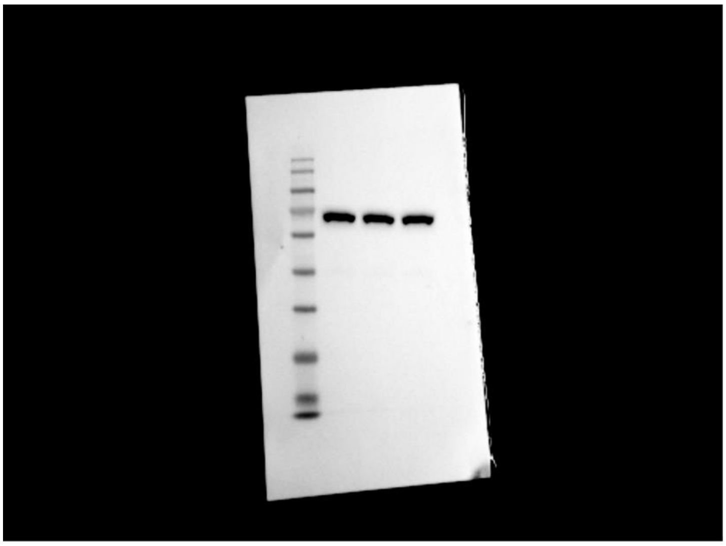

Figure 5B-4

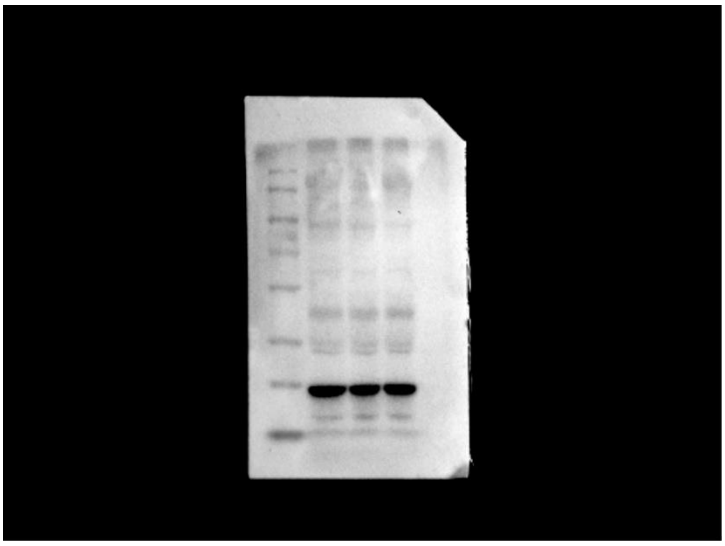

Figure 5B-5

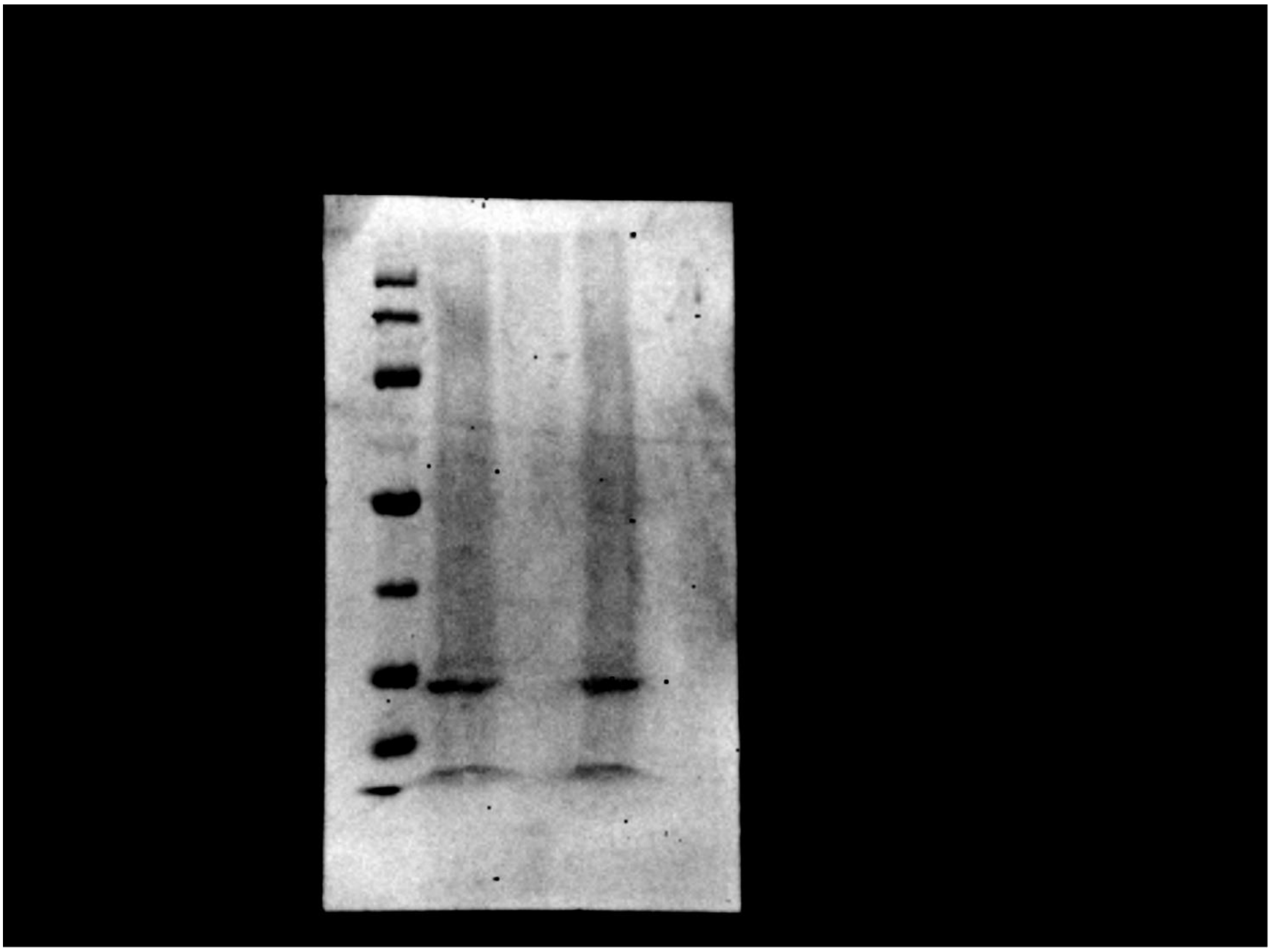

Figure 5C-1

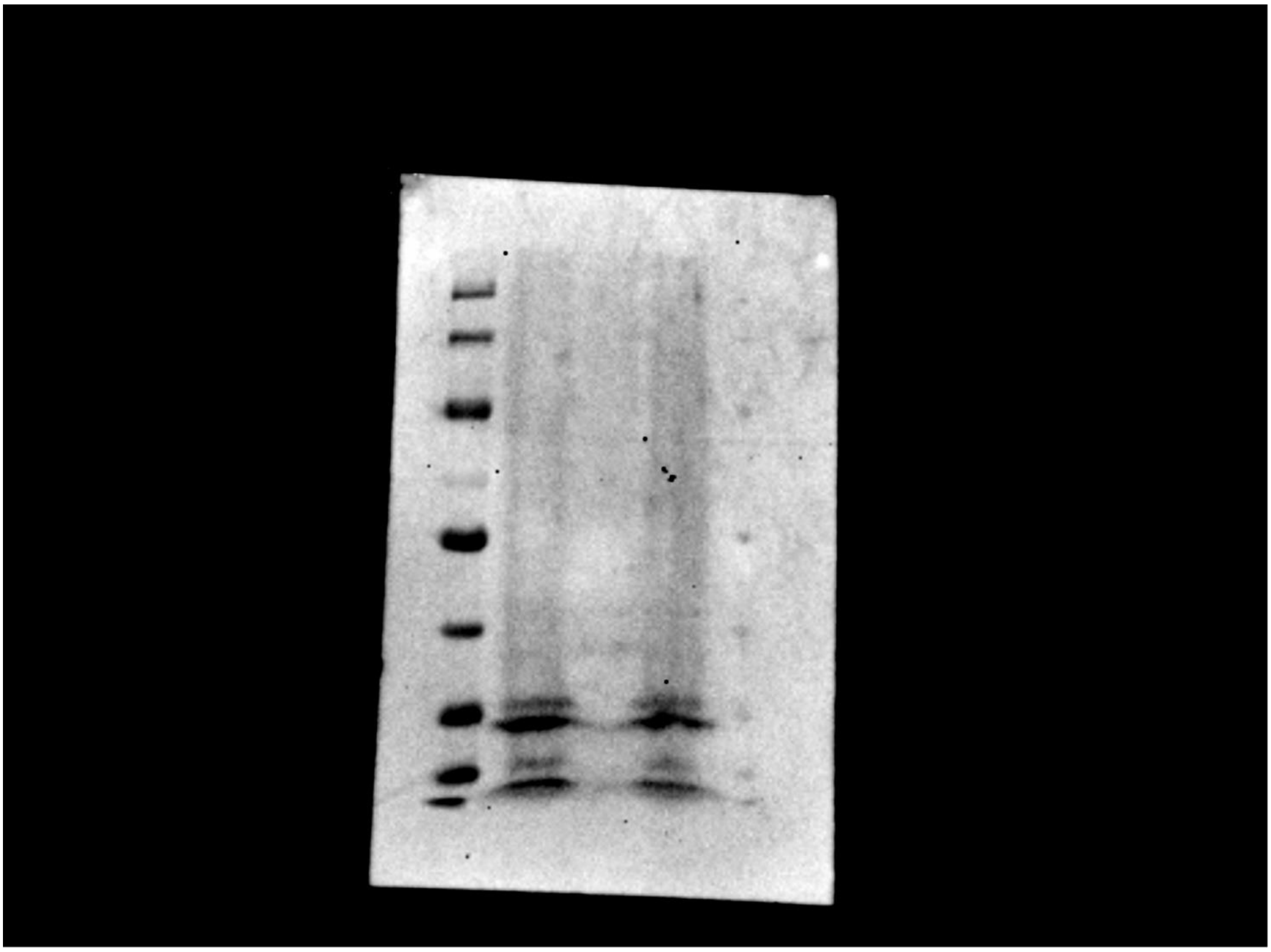

Figure 5C-2

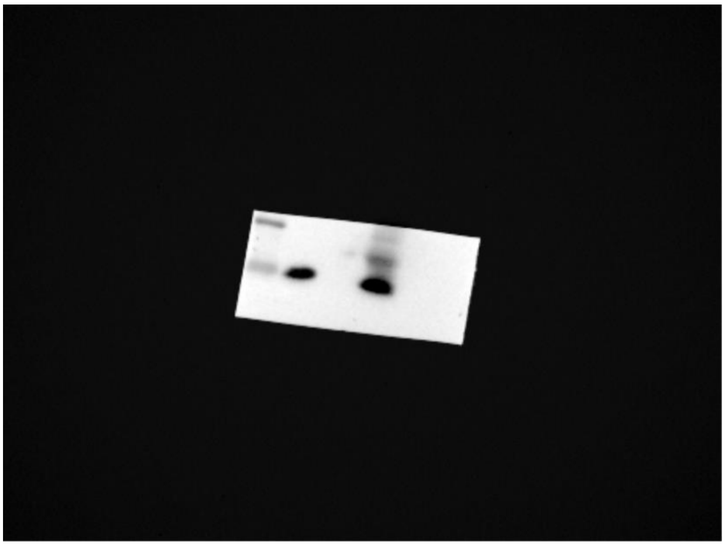

Figure 5C-3

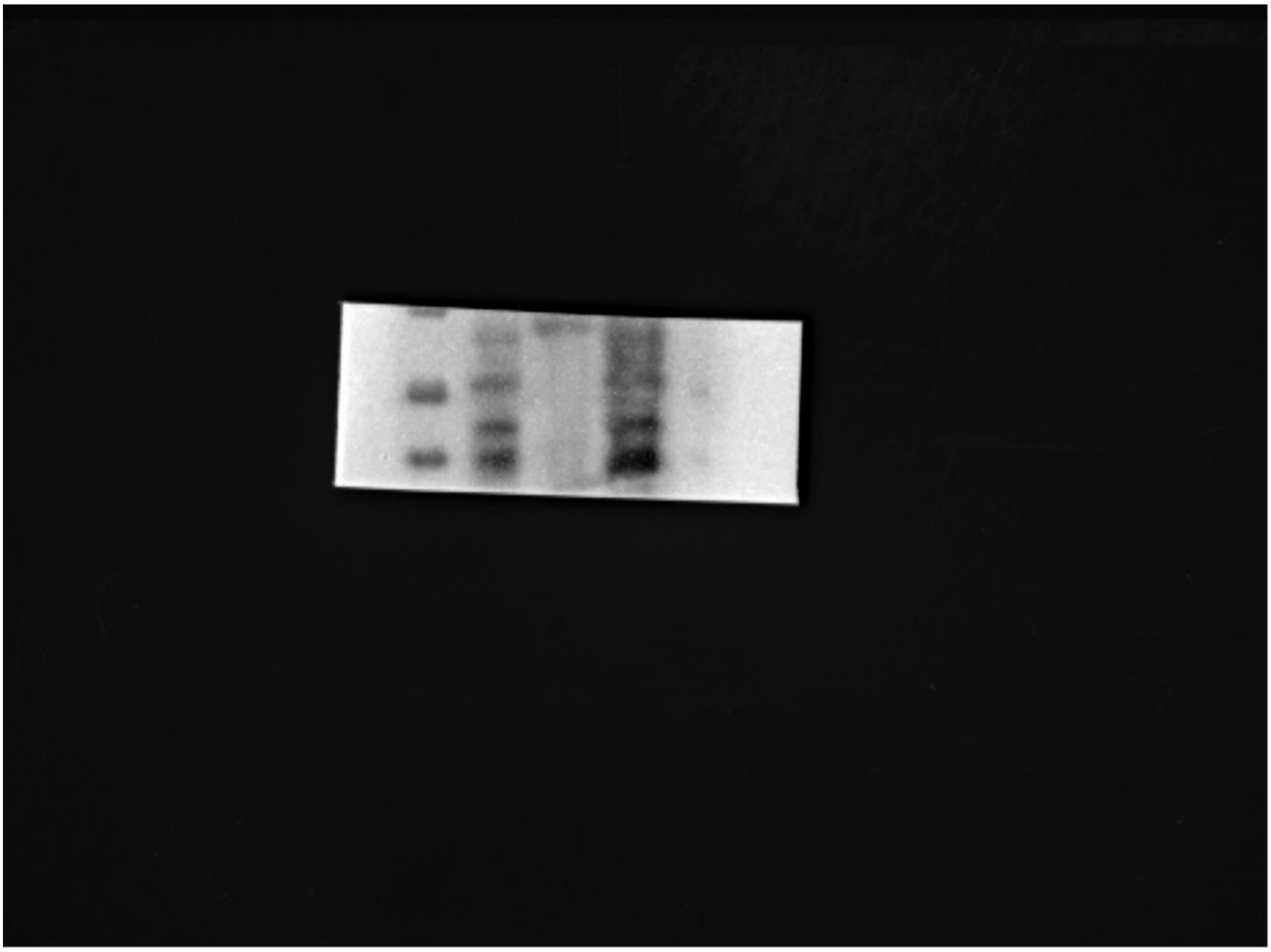

Figure 5C-4

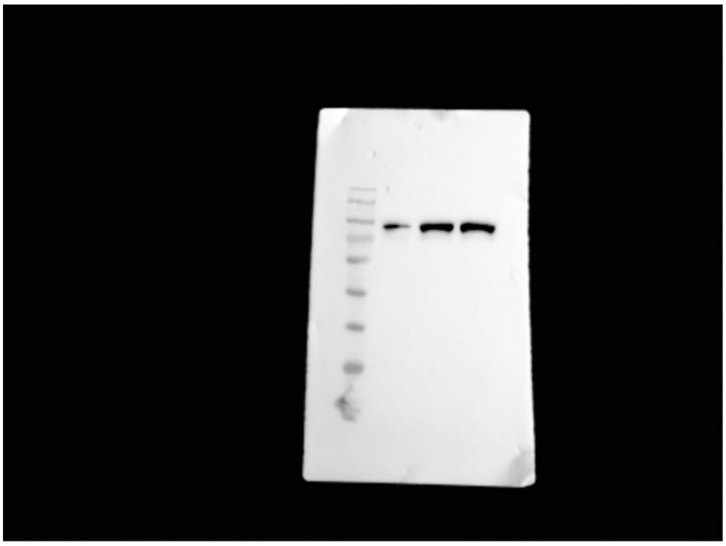

Figure 5D-1

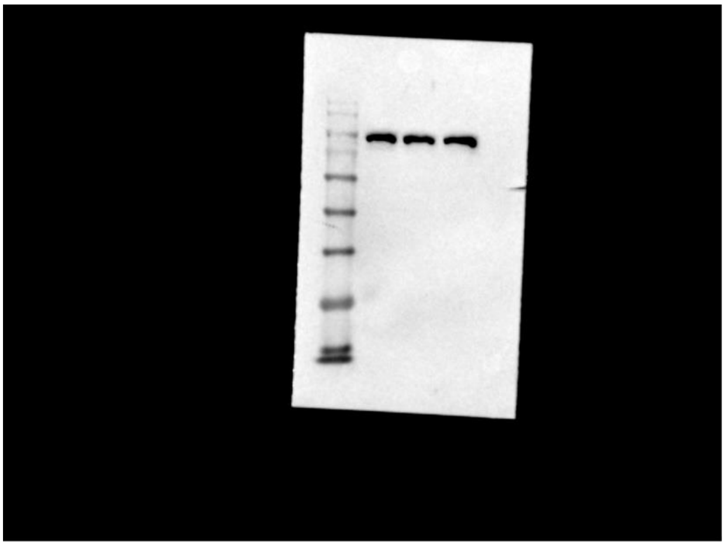

Figure 5D-2

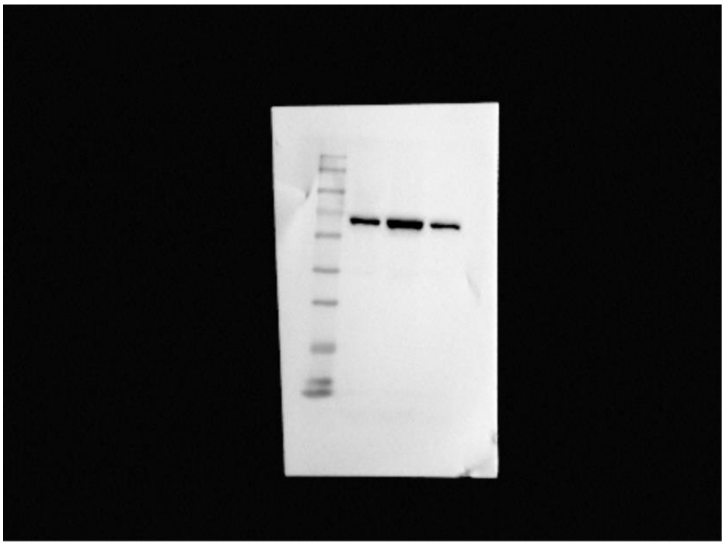

Figure 5D-3

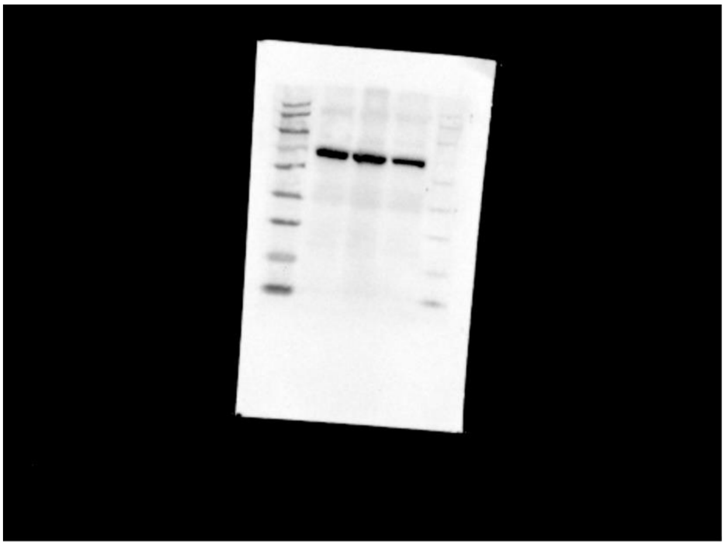

Figure 5D-4

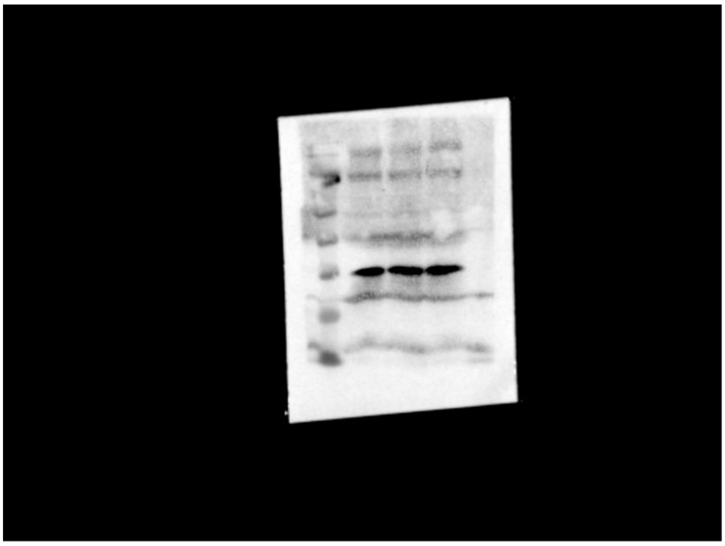

Figure 5D-5

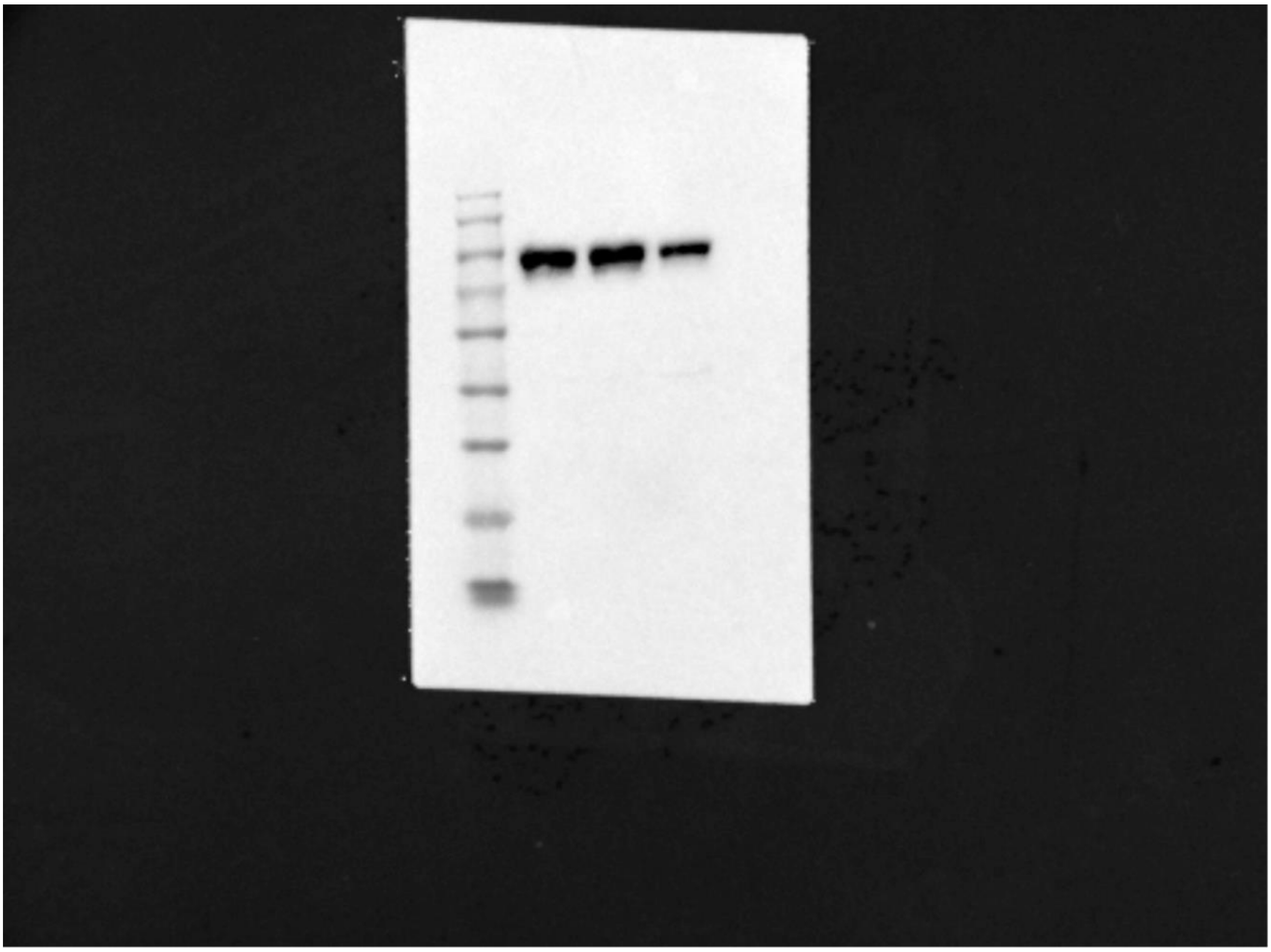

Figure 7D-1

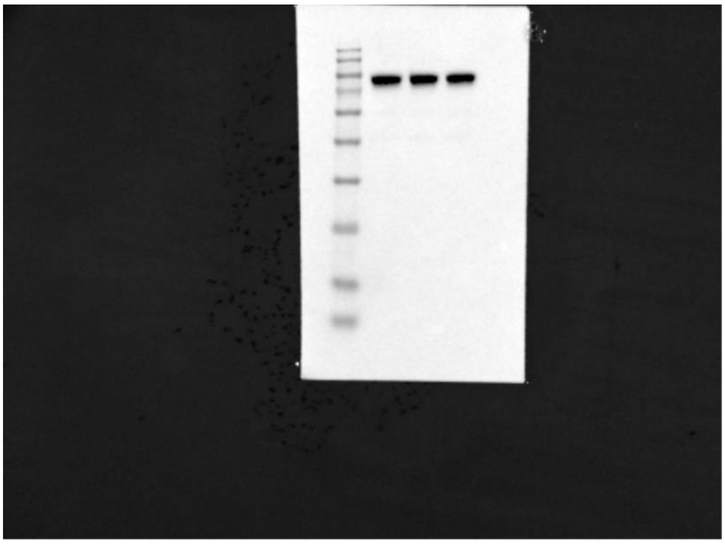

Figure 7D-2

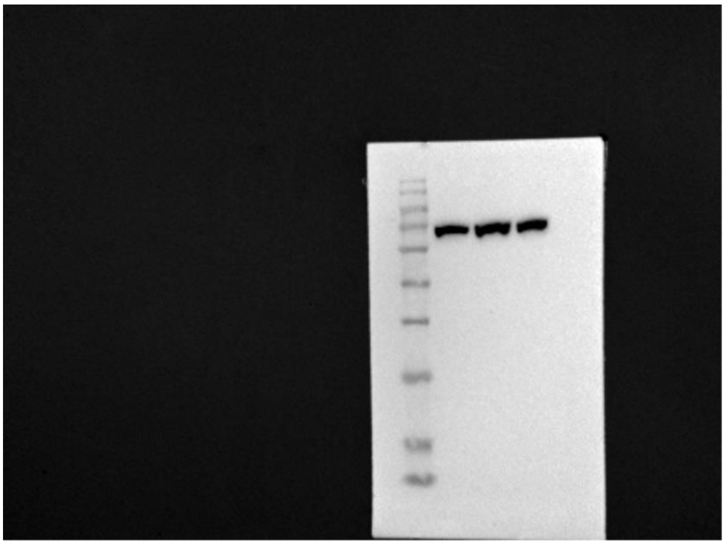

Figure 7D-3

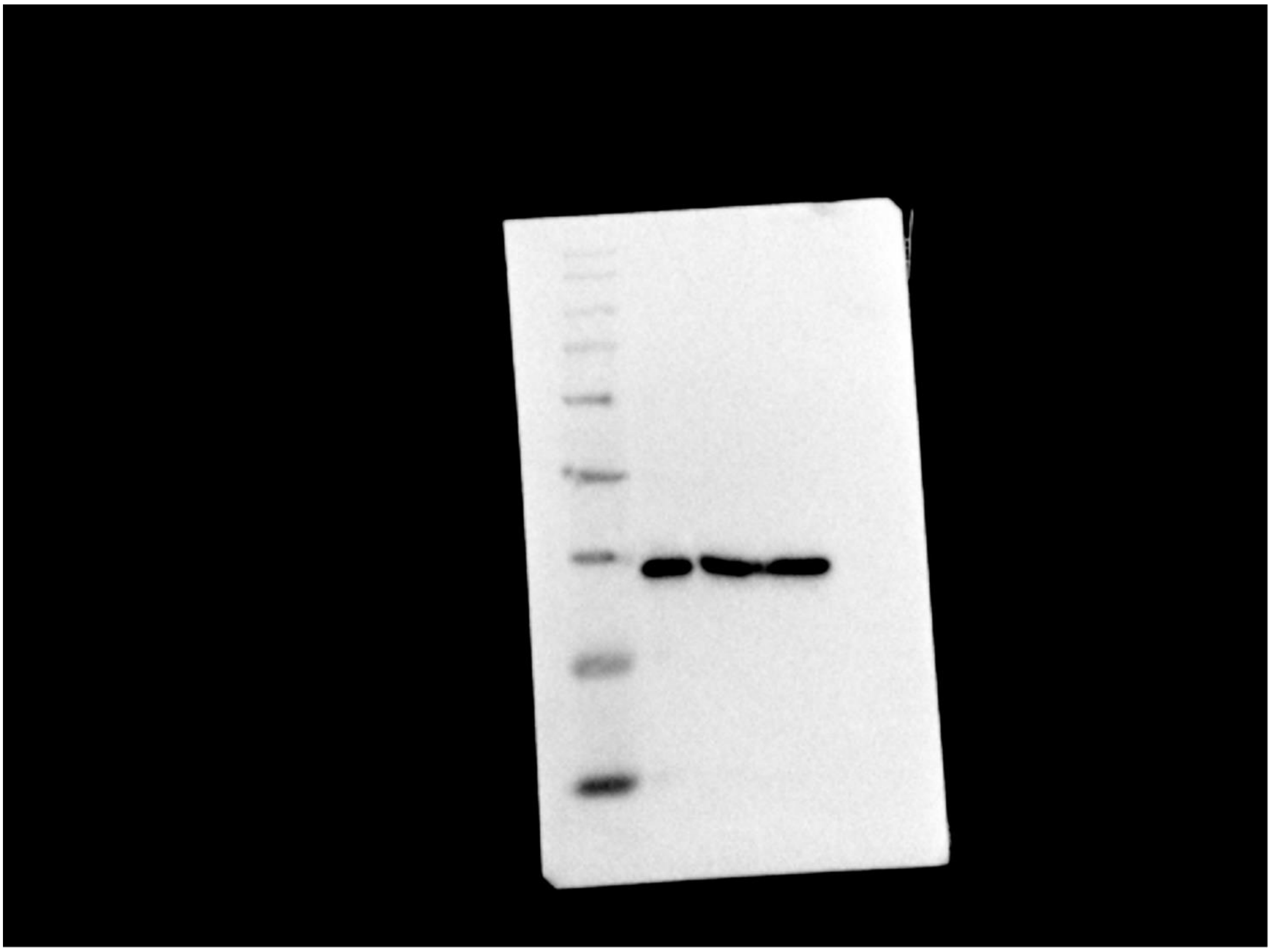

Figure 7D-4
